# Integration of small RNAs, degradome and transcriptome sequencing in hyperaccumulator *Sedum alfredii* uncovers a complex regulatory network and provides insights into cadmium phytoremediation

**DOI:** 10.1111/pbi.12512

**Published:** 2016-01-23

**Authors:** Xiaojiao Han, Hengfu Yin, Xixi Song, Yunxing Zhang, Mingying Liu, Jiang Sang, Jing jiang, Jihong Li, Renying Zhuo

**Affiliations:** ^1^ State Key Laboratory of Tree Genetics and BreedingChinese Academy of ForestryBeijingChina; ^2^ Key Laboratory of Tree Breeding of Zhejiang ProvinceThe Research Institute of Subtropical of ForestryChinese Academy of ForestryHangzhouZhejiangChina; ^3^ Key Laboratory of Agricultural Ecology and EnvironmentCollege of ForestryShandong Agricultural UniversityTai'anShandongChina

**Keywords:** *Sedum alfredii* Hance, cadmium stress, phytoremediation, integration analysis, coexpression network

## Abstract

The hyperaccumulating ecotype of *Sedum alfredii* Hance is a cadmium (Cd)/zinc/lead co‐hyperaccumulating species of Crassulaceae. It is a promising phytoremediation candidate accumulating substantial heavy metal ions without obvious signs of poisoning. However, few studies have focused on the regulatory roles of miRNAs and their targets in the hyperaccumulating ecotype of *S. alfredii*. Here, we combined analyses of the transcriptomics, sRNAs and the degradome to generate a comprehensive resource focused on identifying key regulatory miRNA‐target circuits under Cd stress. A total of 87 721 unigenes and 356 miRNAs were identified by deep sequencing, and 79 miRNAs were differentially expressed under Cd stress. Furthermore, 754 target genes of 194 miRNAs were validated by degradome sequencing. A gene ontology (GO) enrichment analysis of differential miRNA targets revealed that auxin, redox‐related secondary metabolism and metal transport pathways responded to Cd stress. An integrated analysis uncovered 39 pairs of miRNA targets that displayed negatively correlated expression profiles. Ten miRNA‐target pairs also exhibited negative correlations according to a real‐time quantitative PCR analysis. Moreover, a coexpression regulatory network was constructed based on profiles of differentially expressed genes. Two hub genes, *ARF4* (auxin response factor 4) and *AAP3* (amino acid permease 3), which might play central roles in the regulation of Cd‐responsive genes, were uncovered. These results suggest that comprehensive analyses of the transcriptomics, sRNAs and the degradome provided a useful platform for investigating Cd hyperaccumulation in *S. alfredii*, and may provide new insights into the genetic engineering of phytoremediation.

## Introduction

With the food safety issues caused by heavy metals being highly emphasized worldwide, more and more intensive studies on the decontamination of polluted environments have been carried out in recent years (Järup, 2003; Schmitt, 2009). Among the several types of ubiquitous heavy metal pollutants, cadmium (Cd) is known as a harmful agent, which is toxic to all living cells by interfering with DNA repair machines, generating reactive oxygen species (ROS) and disturbing regular gene expression (Jomova and Valko, 2011; Valko *et al*., 2005). In most edible plants, Cd, as a nonessential element, can be competitively accumulated against the uptake of some essential metals, impeding vegetative growth and decreasing crop quality (Seth *et al*., 2008; Varotto *et al*., 2013). However, a special kind of plant, called hyperaccumulating, evolved strategies not only for extreme tolerance to Cd stress but also for hyperaccumulating Cd ions from polluted soils into its above‐ground component (Verbruggen *et al*., 2009). Taking advantage of these plants for the remediation of heavy metals in contaminated soils, namely phytoremediation, has been regarded as an environmentally friendly and economical method (Lombi *et al*., 2001). Among the more than 400 identified hyperaccumulating plants, the hyperaccumulating ecotype of *Sedum alfredii* Hance is a Cd/zinc/lead co‐hyperaccumulating species of Crassulaceae in China (Yang *et al*., 2002). Possessing a high‐performance cellular detoxification system, this plant is a promising phytoremediation candidate that accumulates substantial heavy metal ions in shoots without obvious signs of poisoning (Yang *et al*., 2006). However, more studies at the molecular level are needed to illustrate the mechanism underlying its hyperaccumulating ability.

Cd toxicity in plants can indirectly lead to the overproduction of ROS, such as hydrogen peroxide (H_2_O_2_), superoxide (O_2_˙^−^) and hydroxyl radical (OH˙) (Gill and Tuteja, 2010; Jaspers and Kangasjärvi, 2010), and subsequently causes lipid peroxidation, enzyme inactivation, cell membrane integrity damage, ion leakage and eventually cell death (Boominathan and Doran, 2003). The biochemical efficient enzymatic and nonenzymatic antioxidant defence systems protect plant cells from oxidative stress damages by preventing dangerous elevations of ROS levels (Gill and Tuteja, 2010; Petrov *et al*., 2015). Additionally, the increased ROS concentrations may induce plant programmed cell death (PCD) when the plants were exposed to Cd (Arasimowicz‐Jelonek *et al*., 2012). Plants exposed to Cd can also induce signalling perception through an external signal, and the signal is rapidly transmitted to the responsible transcription factors (Lin and Aarts, 2012). Plant hormones together with some signalling molecules can regulate plant PCD (Gadjev *et al*., 2008). Thus, plants regulate the response to Cd exposure through a complex mechanism.

In recent years, the ever‐accelerated updating of sequencing platforms has made it possible to conduct a large‐scale association analysis of both mRNAs and small RNAs (sRNAs) in nonmodel plants (Morozova and Marra, 2008; Mutz *et al*., 2013). Although the whole transcriptome sequencing of the hyperaccumulating ecotype of *S. alfredii* was described in previous research (Gao *et al*., 2013), a more detailed comparative transcriptome analysis among different Cd stress durations was required to further elucidate the mechanism underlying its hyperaccumulation and hypertolerance of Cd. In the past few years, research has suggested that plant miRNAs and their targets play important regulatory roles in their adaptation to different heavy metal stresses (Gupta *et al*., 2014; Yang, 2013). High‐throughput analysis of these endogenous conserved miRNAs under heavy metal stress might provide a new viewpoint on stress‐response mechanisms in plants (Hsieh *et al*., 2009). At present, many potential heavy metal‐responsive miRNA candidates were identified in rice, medicago, *Brassica juncea*, radish and many other plants (Liu and Zhang, 2012; Wang *et al*., 2015; Zhou *et al*., 2012a,b). A number of miRNAs regulated by aluminium stress were detected in wild soya bean (Zeng *et al*., 2012). *Brassica napus* miRNAs and their targets were profiled on a genomewide scale in response to Cd (Zhou *et al*., 2012a). Furthermore, a genomewide identification of radish miRNAs and their targets was conducted under the stress of Pb exposure (Wang *et al*., 2015). However, little is known regarding miRNA expression profiles in *S. alfredii* in response to heavy metal stress. Therefore, there is also a need for a comprehensive identification of miRNAs involved in heavy metal absorption and resistance in the hyperaccumulating ecotype of *S. alfredii*.

The objective of this study is to systematically identify potential heavy metal‐responsive miRNAs and their targets in the hyperaccumulating ecotype of *S. alfredii*. Thus, the combination of the transcriptomics, sRNAs and degradome generated a comprehensive resource focused on identifying key regulatory miRNA‐targeted circuits under Cd stress. Our results will provide valuable information regarding Cd‐responsive mechanism in the hyperaccumulating ecotype of *S. alfredii*, and help to improve the genetic engineering of phytoremediation.

## Results

### Transcriptome sequencing in hyperaccumulating *S. alfredii* under Cd treatments

The transcriptome library was constructed from mixed RNA pools consisting of root, stem and leaf samples from plants treated with 400 μm CdCl_2_ for eight durations. Then, 44 495 628 raw reads were generated, accounting for approximately 8.8 Gb of sequencing data. After quality control, the remaining high‐quality reads were assembled into 87 721 unigenes, having an average length of 586 bp. The summary of the Illumina transcriptome sequencing of *S. alfredii* is shown in Table 1. The unigenes were annotated by sequence alignments to the NCBI nonredundant protein (Nr) database. According to the BLASTX algorithm's results, 59 319 unigenes were detected to have homologs in the Nr database. To capture the gene expression profiles, we performed RNA sequencing on eight samples from the Cd treatments (control, 0.5 h, 6 h, 12 h, 24 h, 48 h, 72 h and 96 h) to identify differentially expressed genes (DEGs).

**Table 1:** Summary of Illumina transcriptome sequencing for *S. alfredii*

Species	*S. alfredii*
Raw reads	44 495 628
Total nucleotides (bp)	8 851 634 796
Number of contigs	4 362 619
Average contig length (bp)	56
Number of transcripts	140 912
Average transcripts length (bp)	766
Number of unigenes	87 604
Average unigenes length (bp)	586
Q20 (%)	100
GC (%)	45.18

### Sequencing and identification of known and novel miRNAs

The small sRNA library was assembled from eight batches of sequencing data sets to increase coverage (Table S1). After removing the low‐quality sequences, 17‐ to 25‐nt‐long sequences were obtained. The size distribution of the unique sRNAs is summarized in Figure S1, and no significant differences were found among the eight libraries. The displayed lengths of these *S. alfredii* sRNAs ranged from 18 to 25 nt, and the 24‐nt sRNAs displayed the highest redundancy size class among all eight libraries.

**Figure 1:**
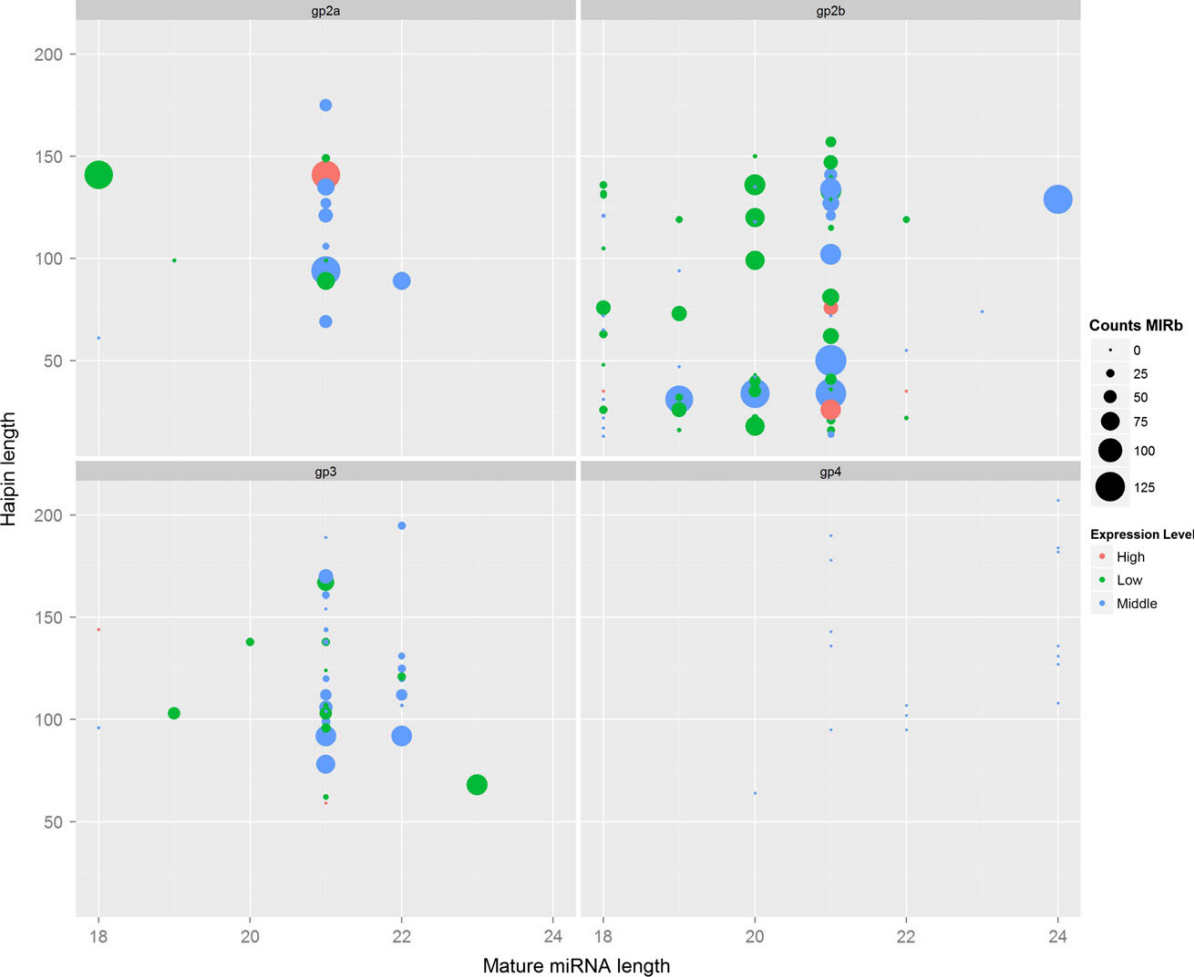
Summary of the distribution of the four identified groups of miRNAs in *Sedum alfredii*. CountsMIRb: the counts of miRNAs from miRBase; Expression level: low indicates <10; middle indicates >10 but less than average; high indicates over average.

All clean and unique sRNAs read sequences were queried using the BLAST algorithm to search against the relevant noncoding RNAs deposited in the Rfam and NCBI GenBank databases to remove rRNA, tRNA, snRNA and snoRNA sequences. To predict novel miRNAs, the remaining unannotated sRNA unique reads that did not match the transcriptome of any library were retrieved and subjected to a secondary structure prediction. All of the loci‐generating sRNAs that could be folded into a secondary structure were considered as potential novel miRNA candidates. In total 356 miRNAs were found in *S. alfredii* and categorized into four different groups based on their abundance in the miRNA database and sequencing reads (Figure 1, Table S2). Of these miRNAs, 260 pre‐miRNAs corresponding to 299 known unique mature miRNAs were identified as having high similarity compared with known plant miRNAs. These miRNAs belonged to 82 already documented miRNA families (Figure S2). The 21 nt length occupied 41.47% of the known unique miRNAs (Table S3). In total, 40 pre‐miRNAs, corresponding to 57 mature miRNAs, were identified as novel miRNA candidates that were not registered in miRBase. The lengths of these miRNAs varied from 18 to 25 nt, with 47.37% being 24 nt long (Table S3). Based on Mfold calculations, the Minimum Free Energy (MFE) and MFE index of these predicted pre‐miRNAs ranged from −23.9 to −147.1 kcal/mol and 0.9 to 1.12 kcal/mol, respectively (Table S4). These characteristics meet the requirements to maintain the stability of the hairpin structures of miRNAs. To further identify authentic miRNAs without a reference in the *Sedum* genome, the secondary structures of pre‐miRNAs and the reads coverage in mature and star areas were analysed. We found 128 highly confident miRNAs (Table S5), of which 30 miRNAs were not found in other plant species.

### Cd‐responsive miRNAs in *S. alfredii*

**Figure 2:**
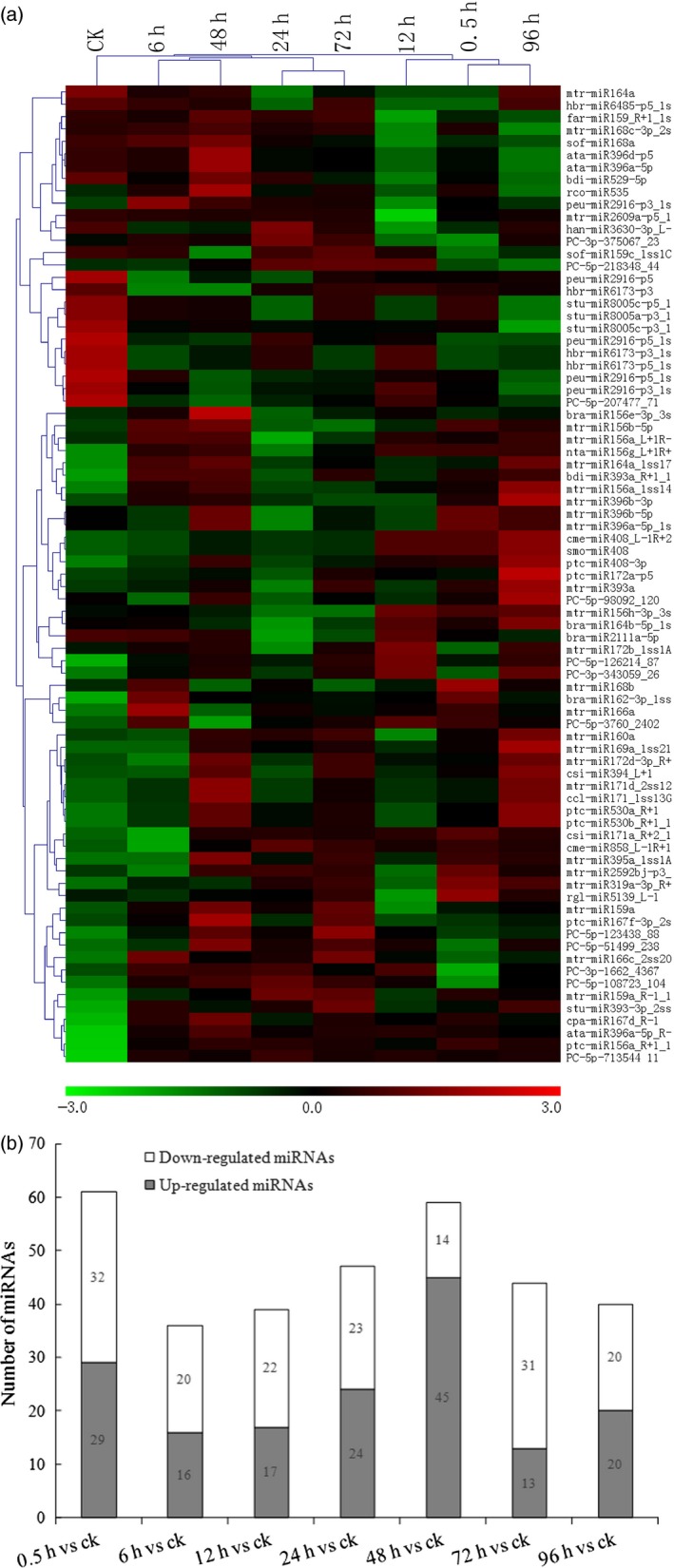
Cadmium (Cd)‐responsive miRNAs in *Sedum alfredii*. (a) Differential expressed miRNAs in eight different Cd treatment durations (0 h, 0.5 h, 6 h, 12 h, 24 h, 48 h, 72 h and 96 h) by hierarchical clustering. Red indicates higher levels of miRNAs and green indicates lower levels of miRNAs. The names of the samples are shown at the top. The original expression values of the miRNAs were normalized using Z‐score normalization. The absolute signal intensity ranges from −3.0 to +3.0, with corresponding colour changes from green to red. (b) The number of differentially expressed miRNAs under Cd stress compared with the control.

To identify miRNAs in *S. alfredii* that respond to Cd, the differential expression of miRNAs in the eight libraries was analysed and compared using the read counts generated from the high‐throughput sequencing. In total, 153 miRNAs (*P‐*value <0.05) showed differential expression patterns. When a stricter criterion of total expression abundance >10 was used, 79 differentially expressed miRNAs, including 67 known miRNAs and 12 novel miRNAs, were found among the eight libraries. The heatmap of potential differentially expressed miRNAs is illustrated in Figure 2a. Most miRNA members of the same family had similar expression profiles. For example, three *miR408* and three *miR171* family members were significantly up‐regulated by Cd exposure. Five *miR2916*, three *miR6173* and three *miR8005* family members were found to be significantly down‐regulated by Cd treatments. Among the 12 novel miRNAs, 10 miRNAs were up‐regulated by Cd exposure, while the other two miRNAs (*PC‐5p‐98092_120* and *PC‐5p‐207477_71*) were down‐regulated (Figure 2a).

In addition to the validation of differentially expressed miRNAs, we also examined the distribution of 153 differential miRNAs between control and treatments (Figure 2b). Among these miRNAs, the largest number (45 miRNAs) of up‐regulated miRNAs was found with a CdCl_2_ treatment of 48 h compared with the control, while 32 miRNAs were significantly down‐regulated with a CdCl_2_ treatment of 0.5 h compared with the control (Figure 2b). These data indicated that the duration of Cd treatments might cause short‐term as well as long‐term effects on the expression levels of miRNAs.

### Target prediction of the known and novel miRNAs by degradome sequencing

Through degradome sequencing, 9 649 268 raw reads representing 4 721 650 unique reads, were generated from the mixed degradome pools. After removing the reads lacking the adaptor, 6 812 869 (70.6% of all reads) sequences were successfully mapped to the 48 446 unigenes (55.22% of the 87 721 input cDNA sequences) of the hyperaccumulating *S. alfredii*. The CleaveLand 3.0 pipeline was used to identify degraded targets for each of the miRNA families (Addo‐Quaye *et al*., 2009). All of the potential cleaved transcripts were classified into five categories, based on the signature abundance at each occupied transcript position (Xu *et al*., 2013; Yang *et al*., 2013): category 0—over one raw read at the position, abundance at the position is equal to the maximum on the transcript, and there is only one maximum on the transcript; category 1—over one raw read at the position, abundance at the position is equal to the maximum on the transcript, and there is more than one maximum position on the transcript; category 2—over one raw read at the position, abundance at position is less than the maximum but higher than the median for the transcript; category 3—over one raw read at the position, abundance at the position is equal to or less than the median for the transcript; and category 4—only one raw read at the position. Among these identified targets, there were 171, 16, 216, 8 and 346 in categories 0, 1, 2, 3 and 4 respectively (Table S6, Figure S3).

A total of 722 targets for 180 known miRNAs were identified from the mixed degradome (Table S6). We also identified the targets of 57 novel newly discovered miRNAs by degradome sequencing and analysis. Finally only 14 novel miRNAs targeted 32 transcripts (Table 2). Most of the miRNAs (187) cleaved a single transcript target, while 29 miRNAs were detected to cleave two or more different transcript targets (Table S6). Forty‐seven targets were identified to be cleaved by *ptc‐miR171i‐p3_1ss21TG*, which was the highest amount of transcripts cleaved by the same miRNA; while 11 miRNAs were detected to cleave only one transcript target, which was annotated to an unknown protein with hydrolase activity.

**Table 2:** Target genes of 14 novel miRNAs and their functional annotation

Small RNA	Targets	Alignment Score	Cleavage Site	Category	Target annotation	Biological process
PC‐3p‐1507684_5	BMK. 61478	3	1247	4	GPI‐anchored protein	Anchored to plasma membrane
PC‐3p‐1650016_5	BMK. 62314	3.5	2225	4	Unknown	‐
PC‐3p‐1662_4367	BMK. 83121	4	259	2	Unknown	‐
PC‐3p‐3238323_4	BMK. 53452	2.5	992	4	GRF1‐interacting factor 3	Regulation of transcription,
	BMK. 62570	2.5	59	2	Class III HD‐Zip protein 8	Regulation of transcription
PC‐3p‐343059_26	BMK. 62457	3	819	0	Homeobox‐leucine zipper protein REVOLUTA‐like	Regulation of transcription
	BMK. 21295	4	304	4	Unknown	‐
	BMK. 51337	3	2136	2	Retrotransposon protein	‐
	BMK. 61478	0	1233	4	GPI‐anchored protein	Anchored to plasma membrane
PC‐3p‐3461203_2	BMK. 36956	3.5	1616	4	Leucine‐rich repeat receptor‐like protein kinase (LRR‐RLK)	Protein phosphorylation
	BMK. 39438	3.5	1163	4	Cytochrome P450	Secondary metabolites biosynthesis
	BMK. 46596	3.5	1907	2	Subtilisin‐like protease	Proteolysis
	BMK. 48392	4	2113	4	Nitrate transporter	Oligopeptide transport
	BMK. 49292	4	1691	4	Run and tbc1 domain containing 3	Regulation of Rab GTPase activity
	BMK. 52038	4	1970	4	Alpha‐glucan water dikinase	Starch catabolic process
	BMK. 52079	4	2014	4	Serine/threonine protein phosphatase 2A	Signal transduction
	BMK. 59928	4	1455	4	Translocase subunit SecA	Protein targeting
	BMK. 61399	3.5	2024	4	Transcription factor APETALA2	Regulation of transcription
	BMK. 83033	4	948	4	Lyrata myb family transcription factor	Regulation of transcription
PC‐5p‐123438_88	BMK. 51365	4	969	4	Sentrin‐specific protease	Nuclear‐transcribed mRNA catabolic process
PC‐5p‐126214_87	BMK. 52369	4	287	2	Unknown	‐
PC‐5p‐20273079_1	BMK. 49984	2	170	2	Unknown	‐
	BMK. 51107	4	1355	1	Unknown	‐
	BMK. 56528	3.5	987	4	Receptor‐like cytosolic serine/threonine protein kinase RBK2‐like	Protein phosphorylation
	BMK. 85664	3.5	385	4	Galacturonosyltransferase‐like 9‐like	Response to oxidative stress
PC‐5p‐207477_71	BMK. 35227	3	1258	4	Polyamine oxidase	Oxidation–reduction process
	BMK. 40236	4	662	4	Unknown	‐
PC‐5p‐2679901_5	BMK. 61478	2	1326	0	GPI‐anchored protein	Anchored to plasma membrane
PC‐5p‐3760_2402	BMK. 50544	1	1587	2	Auxin response factor 4	Response to hormone stimulus
	BMK. 58380	0	1763	2	Auxin response factor 3	Response to hormone stimulus
PC‐5p‐51499_238	BMK. 61478	0	1326	0	GPI‐anchored protein	Anchored to plasma membrane
PC‐5p‐6279784_1	BMK. 62367	4	1505	4	Unknown	‐

### Annotation and enrichment analysis of targets for miRNAs under cadmium stress

**Figure 3:**
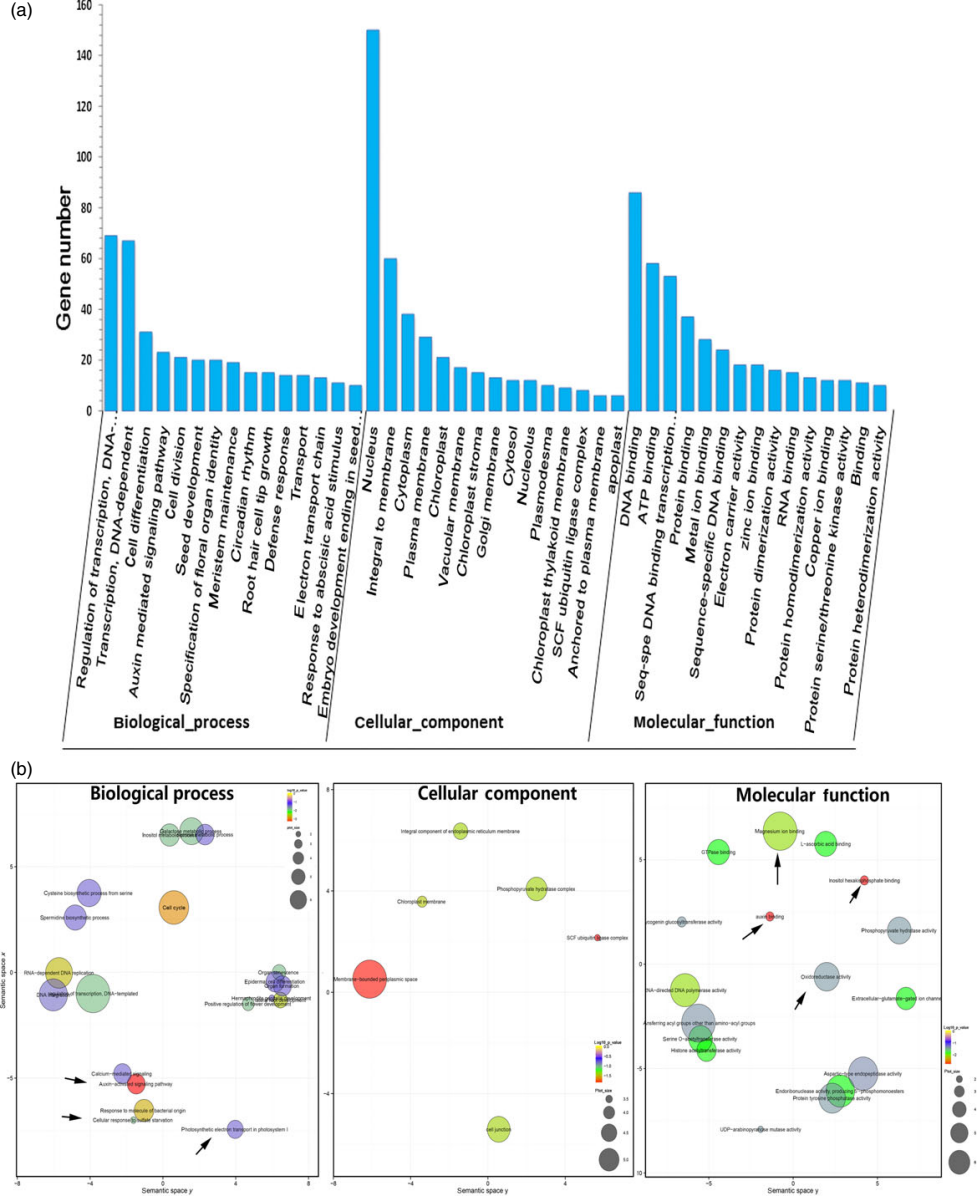
Gene ontology (GO) functional classification of identified target genes. (a) Gene ontology classification of target genes for the identified miRNAs, (b) GO enrichment of target genes. The arrows represent the important biological pathways.

An analysis using the BLASTX algorithm showed that the identified target transcripts detected in our degradome library shared homology with other plant proteins. A gene ontology (GO) functional classification analysis was conducted to further understand the functions of these 754 identified targeted genes (Figure 3a). These target genes predominantly participated in biological processes, cellular component and molecular function. Fifteen transcripts were identified in biological processes, with the two most frequent categories being ‘regulation of transcription’ and ‘transcription’. Of the 15 molecular component categories, the two most highly represented were ‘nucleus’ and ‘integral to membrane’. Finally, there were 15 molecular function categories, with the most abundant being ‘DNA binding’ (Figure 3a). The functional annotations indicated that there were five transcription factors, *NAC82*,* NAC90*,* HD‐Zip III*,* AP2‐like ethylene‐responsive transcription factor* and *Auxin signalling F‐box 2*, that responded to various stresses (Krishnamurthy and Rathinasabapathi, 2013; Naika *et al*., 2013). Another four targets, *Squamosa promoter binding protein‐like 12*,* Squamosa promoter binding protein‐like 3*,* Senescence‐associated protein DIN1* and *ATP sulphurylase 1*, had functions related to heavy metal stress (Contreras‐Porcia *et al*., 2011; Perea‐García *et al*., 2010; Wangeline *et al*., 2004). This analysis suggested that the miRNA targets were concentrated in the transcription factors and respond to stimuli.

An analysis of the GO term enrichment indicated that key biological pathways were involved in the Cd responses. Significantly enriched GO terms (Fisher's test *P‐*value <0.05) were visualized using the reviGO tool (Supek *et al*., 2011) and plotted (Figure 3b). Some interesting GO terms related to metal transport (GO: 0000287 magnesium ion binding and GO: 0005234 extracellular‐glutamate‐gated ion channel activity), redox‐related secondary metabolism (GO: 0016706 oxidoreductase activity, GO: 0000822 inositol hexakisphosphate binding, GO: 0008295 spermidine biosynthetic process), and phytohormone auxin (GO: 009734, GO: 0010011 auxin binding) were revealed, which was in agreement with our current understanding of Cd responses.

### Correlation analysis of miRNAs expression profiles and their target genes

**Figure 4:**
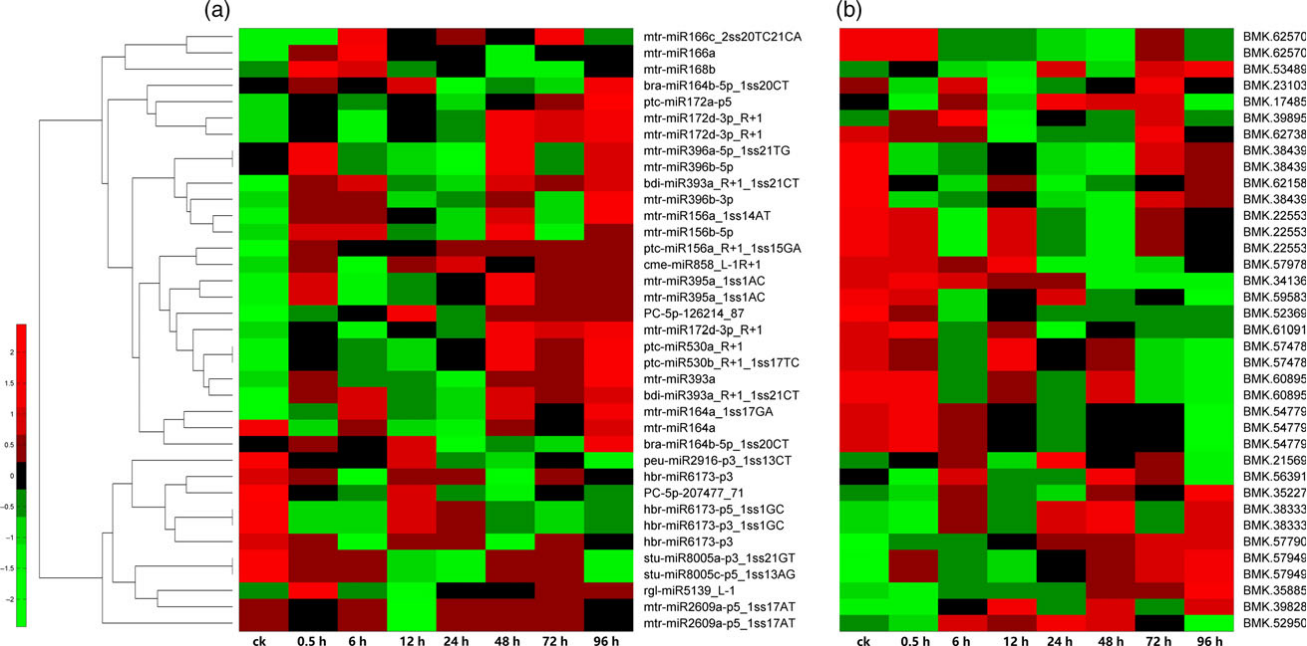
A combined view of expressions levels between differentially expressed miRNAs (a) and their target genes (b) in *Sedum alfredii* at eight different Cd treatment durations. The original expression values of miRNAs and their target genes were normalized by Z‐score normalization.

To investigate the variable trend of the global transcriptome in the hyperaccumulating *S. alfredii* under different Cd stress treatment durations, the previously assembled unigenes were applied to a DEG analysis. In total, 342 target genes presented differential expression levels under Cd stress. There were 45 differentially expressed targets from 40 miRNAs (Table 3) when the targets corresponded to differentially expressed miRNAs, and 39 miRNA‐target pairs showed a reverse expression pattern (negatively correlated; Figure 4). For example, *cme‐miR858_L‐1R+1* was significantly down‐regulated by Cd exposure, while its target *MYB12* (BMK. 57978) was significantly up‐regulated.

**Table 3:** The differentially expressed targets of Cd‐responsive differentially expressed miRNAs

miRNA Family	miRNA name	Targets	Annotation
miR156	mtr‐miR156b‐5p	BMK. 22553	Squamosa promoter binding protein‐like 12
	ptc‐miR156a_R + 1_1ss15GA	BMK. 22553	Squamosa promoter binding protein‐like 12
		BMK. 31642	Squamosa promoter binding protein‐like 3
	mtr‐miR156a_1ss14AT	BMK. 22553	Squamosa promoter binding protein‐like 12
		BMK. 31642	Squamosa promoter binding protein‐like 3
miR164	bra‐miR164b‐5p_1ss20CT	BMK. 23103	NAC domain‐containing protein 80
		BMK. 54779	NAC domain‐containing protein 92
	mtr‐miR164a	BMK. 23103	NAC domain‐containing protein 80
		BMK. 54779	NAC domain‐containing protein 92
	mtr‐miR164a_1ss17GA	BMK. 23103	NAC domain‐containing protein 80
		BMK. 54779	NAC domain‐containing protein 92
miR166	mtr‐miR166a	BMK. 62570	HD‐Zip III
	mtr‐miR166c_2ss20TC21CA	BMK. 62570	HD‐Zip III
miR167	cpa‐miR167d_R‐1	BMK. 47519	Senescence‐associated protein DIN1
miR168	mtr‐miR168b	BMK. 53489	Argonaute 1
miR172	ptc‐miR172a‐p5	BMK. 17485	S‐adenosyl‐L‐methionine‐dependent methyltransferases superfamily protein
		BMK. 58875	Nonphototropic hypocotyl 1
		BMK. 58935	Monoglyceride lipase
	mtr‐miR172d‐3p_R + 1	BMK. 39895	Two‐component response regulator‐like APRR2
		BMK. 61091	AP2‐like ethylene‐responsive transcription factor
		BMK. 61399	Floral homeotic protein APETALA 2
		BMK. 62738	Pre‐mRNA‐splicing factor CWC22
	mtr‐miR172b_1ss1AG	BMK. 61091	AP2‐like ethylene‐responsive transcription factor TOE3
		BMK. 61399	Floral homeotic protein APETALA 2
miR393	mtr‐miR393a	BMK. 60895	Auxin signalling F‐box 2
		BMK. 62158	Unknown
	bdi‐miR393a_R + 1_1ss21CT	BMK. 60895	Auxin signalling F‐box 2
		BMK. 62158	Unknown
miR394	csi‐miR394_L + 1	BMK. 39348	Mediator of RNA polymerase II transcription subunit 37a
miR395	mtr‐miR395a_1ss1AC	BMK. 34136	Unknown
		BMK. 59583	sulphate transporter 2.1
		BMK. 61906	ATP sulphurylase 1
miR396	mtr‐miR396b‐5p	BMK. 38439	Unknown protein
		BMK. 63219	KRR1 small subunit processome component homolog
	mtr‐miR396a‐5p_1ss21TG	BMK. 38439	Unknown
		BMK. 63219	KRR1 small subunit processome component homolog
	mtr‐miR396b‐3p	BMK. 38439	Unknown protein
		BMK. 63219	KRR1 small subunit processome component homolog
miR408	cme‐miR408_L‐1R+2	BMK. 85914	Basic blue copper protein
	smo‐miR408	BMK. 85914	Basic blue copper protein
	ptc‐miR408‐3p	BMK. 85914	Basic blue copper protein
miR530	ptc‐miR530a_R + 1	BMK. 57478	Unknown
	ptc‐miR530b_R + 1_1ss17TC	BMK. 57478	Unknown
miR858	cme‐miR858_L‐1R+1	BMK. 57978	MYB12
miR2609	mtr‐miR2609a‐p5_1ss17AT	BMK. 21051	RING finger and CHY zinc finger domain‐containing protein 1
		BMK. 39828	Fructose‐1,6‐bisphosphatase
		BMK. 52950	Glutathione S‐transferase U17‐like
		BMK. 59655	Disulphide isomerase
		BMK. 83779	Glycerol‐3‐phosphate acyltransferase
miR2916	peu‐miR2916‐p3_1ss13CT	BMK. 21569	UDP‐glucose transporter 3
miR3630	han‐miR3630‐3p_L‐2R‐1_1ss21TA	BMK. 21215	Amino acid permease 3
miR5139	rgl‐miR5139_L‐1	BMK. 35885	DEAD‐box ATP‐dependent RNA helicase 3
		BMK. 60148	UDP‐glycosyltransferase 75D1‐like
miR6173	hbr‐miR6173‐p3_1ss1GC	BMK. 38333	Calcium‐binding EF‐hand family protein
	hbr‐miR6173‐p5_1ss1GC	BMK. 38333	Calcium‐binding EF‐hand family protein
	hbr‐miR6173‐p3	BMK. 37451	ATP synthase subunit alpha
		BMK. 56391	Probable inactive purple acid phosphatase 27
		BMK. 57790	Aspartic protease in guard cell 1
miR8005	stu‐miR8005c‐p5_1ss13AG	BMK. 45712	Haloacid dehalogenase‐like hydrolase (HAD) superfamily protein
		BMK. 57949	BEL1‐like homeodomain protein 1
	stu‐miR8005a‐p3_1ss21GT	BMK. 45712	Haloacid dehalogenase‐like hydrolase (HAD) superfamily protein
		BMK. 57949	BEL1‐like homeodomain protein 1
	stu‐miR8005c‐p3_1ss12AG	BMK. 45712	Haloacid dehalogenase‐like hydrolase (HAD) superfamily protein
Novel miRNA	PC‐5p‐3760_2402	BMK. 50544	Auxin response factor 4
	PC‐3p‐1662_4367	BMK. 83121	Unknown
	PC‐5p‐126214_87	BMK. 52369	Knob‐associated histidine‐rich protein
	PC‐5p‐207477_71	BMK. 35227	Polyamine oxidase
		BMK. 40236	DNA topoisomerase 1‐like

**Figure 5:**
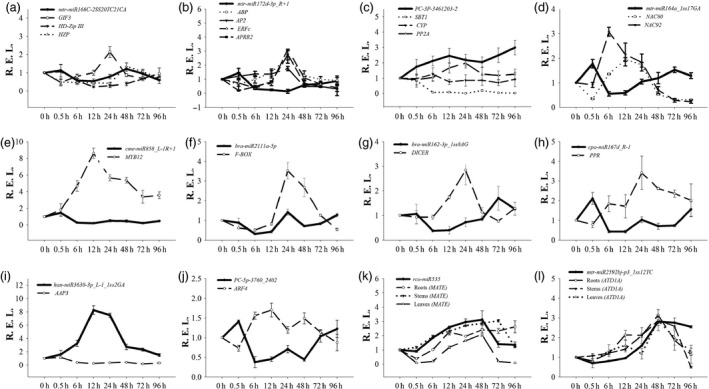
Expression correlation between miRNAs and their targets at eight different Cd treatment durations. (a ) mtr‐miR166C‐2SS20TC21CA; (b) mtr‐miR172d‐3p_R+1; (c) PC‐3P‐3461203‐2; (d) mtr‐miR164a_1ss17GA; (e) cme‐miR858_L‐1R+1; (f) bra‐miR2111a‐5p; (g) bra‐miR162‐3p_1ss8AG; (h) cpa‐miR167d_R‐1; (i) han‐miR3630‐3p_L‐1_1ss2GA; (j) PC‐5p‐3760_2402; (k) rco‐miR535; (l) mtr‐miR2592bj‐p3_1ss12TC. The thick and thin lines indicate miRNAs and accordingly the target abundance, respectively, based on the RT‐qPCR results.

A real‐time quantitative PCR (RT‐qPCR) analysis was also performed to validate the expression profile of 12 interesting miRNA‐target modules. Our results showed that the RT‐qPCR analysis for most of the differentially expressed miRNAs and their targets displayed similar expression pattern to those generated from high‐throughput sequencing (Figure 5). Furthermore, 10 miRNA‐target pairs (*mtr‐miR166C‐2SS20TC21CA/GIF3*,* mtr‐miR172d‐3p_R + 1/ABP/AP2/ERFc/APRR2*,* PC‐3P‐3461203‐2/SBT1/CYP/PP2A*,* mtr‐miR164a_1ss17GA/NAC80/NAC92*,* cme‐miR858_L‐1R+1/MYB12*,* bra‐miR2111a‐5p/F‐BOX*,* ptc‐miR162a_1ss8AG/DICER*,* cpa‐miR167d_R‐1/PPR*,* han‐miR3630‐3p_L‐1_1ss2GA/AAP3*, and *PC‐5p‐3760_2402/ARF4*) exhibited a negative relationship at the expression level, indicating that a transcriptional repression may be mediated on these targets through their corresponding miRNAs (Figure 5a–j). In addition, we detected two other miRNAs (*rco‐miR535* and *mtr‐miR2592bj‐p3_1ss12TC*) and their targets in roots, leaves and stems (Figure 5k–l). The two miRNA‐target pairs presented a positive relationship at the expression level. The expression levels of these genes gradually increased from 0 h to 48 h after Cd stress and then decreased after 48 h.

### Gene coexpression network analysis

To determine the genes related to the coexpressed target genes, the DEG data set was used to construct a network. After excluding nonexpressed and lowly expressed genes, we identified 60 gene coexpression modules containing 31 985 unigenes (Figure S4). Ultimately, the resulting network was composed of 31 distinct gene modules. The constructed gene modules ranged in size from 4238 (turquoise module) to 136 genes (plum1 module). To investigate the network connections for the target genes, we focused on the two target genes *ARF4* (BMK. 50544, auxin response factor 4, *PC‐5P‐3760_2402* target gene) and *AAP3* (BMK. 21215, amino acid permease 3, *han‐miR3630‐3p_L‐2R‐1_1ss21TA* target gene), which play a prominent roles in heavy metal stress (Gielen *et al*., 2012; Xu *et al*., 2012). A complete list of module assignments and network metrics for the two genes is included in Table S7. In this network, the two hub genes *ARF4* and *AAP3* were directly connected with 1754 edges and 1212 edges. Among them, 1115 edges were coregulated by the two hub genes, suggesting that the coexpressed genes were most likely coregulated.

**Figure 6:**
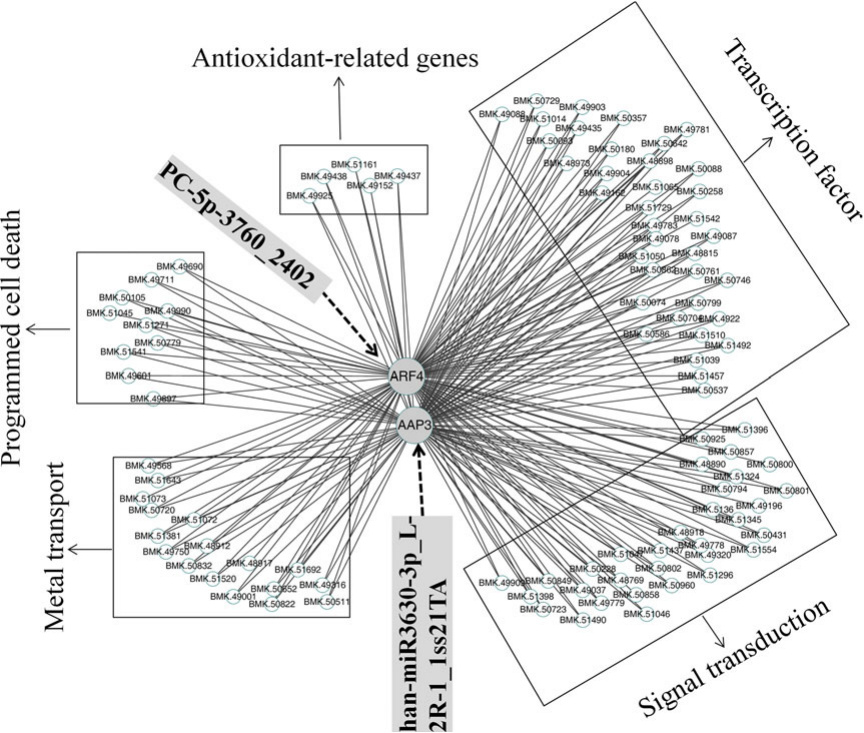
The coexpression subnetwork of *ARF4* and *AAP3*.

We also created a subnetwork containing 91 genes by identifying five categories that were related to signal transduction, transcription factor, antioxidant‐related genes, PCD and metal transport (Figure 6). Of the 91 genes, 32 and 36 were in the signal transduction and transcription factor categories respectively when testing the functional assignments (Table S8). The two categories may trigger transport processes for detoxification, activate biochemical defence reactions, and PCD under Cd stress. Seventeen metal transport‐related genes might be involved in Cd uptake, transport and sequestration processes. Five members of the peroxidase family found in the subnetwork may remove H_2_O_2_ formed because of Cd tress, and lead to the activation of other defence mechanisms. When the plants experience heavy metal tress, plant PCD, as a positive and negative aspect of environment adaptation, provides survival benefits for the whole plant. More importantly, this network can be exploited in future studies to identify novel genes that interact with *ARF4* and *AAP3*.

## Discussion

In comparison with miRNAs identified from the other plants, little research has been conducted on the role of miRNAs in Crassulaceae plants. Only 146 EST sequences of *S. alfredii* have been deposited in GenBank, which is insufficient to analyse miRNA behaviour under Cd stress. Here, a transcriptome data set was used as reference sequence for miRNA and degradome sequencing analyses in *S. alfredii*. The approach highlighted the benefits of providing more qualitative and quantitative descriptions of the miRNA functions during stress tolerance. Furthermore, 356 miRNAs, including 128 highly confident miRNAs, were identified based on the transcriptome data in the hyperaccumulating *S. alfredii*. Of these miRNAs, more than half (68.35%) had relatively low expression abundances, indicating that high‐throughput sequencing is a most powerful strategy to identify miRNAs with low expression levels in plants. Additionally, the sRNA length distribution patterns peaked at 24 nt (Figure S1), which is consistent with previous results for most angiosperms, such as peanut (Zhao *et al*., 2010), soya bean (Song *et al*., 2011), *Medicago truncatula* (Szittya *et al*., 2008), *Paulownia australis* (Niu *et al*., 2014) and cotton (Yang *et al*., 2013). Therefore, the sRNA length distribution pattern in *S. alfredii* is similar to that in other plants.

In this study, we identified 79 miRNAs differentially regulated by Cd exposure (Figure 1). Most miRNA members of the same family showed similar expression profiles. For instance, three *miR396* and three *miR171* family members were significantly up‐regulated by Cd exposure. In *M. truncatula*, it was reported that *miR171* was also up‐regulated by Cd stress (Zhou *et al*., 2008). However, *miR171* in rice was reported to be down‐regulated in response to Cd treatment (Ding *et al*., 2011; Tang *et al*., 2014); *miR171* and *miR396* were also down‐regulated by Cd exposure in *B. napus* (Xie *et al*., 2007; Zhou *et al*., 2012a). Thus, the differential expression patterns of *miR171* and *miR396* might be due to the different Cd treatment conditions of plant species. We also discovered that some previously known miRNAs were regulated by Cd stress. Five *miR2916*, three *miR6173* and three *miR8005* family members were found to be significantly down‐regulated by Cd treatments (Figure 2a). In plants, *miR2916* was found to be responsive to abiotic stresses, such as salinity (Qin *et al*., 2015) and boron stress (Ozhuner *et al*., 2013). In *Saccharina japonica*,* miR6173* was reported to regulate the response to heat stress (Liu *et al*., 2015). These findings implied that the known miRNAs were most likely involved in cross‐adaptation to regulate plant tolerance to abiotic stresses, including heavy metal stress. However, their exact functions remain to be verified in future investigations. Different miRNA members within the same family were present at different expression levels after Cd exposure. Similar events also occured with miRNA family members in other plants. In soya bean, *miR396a‐3p* and *miR396i‐3p* were significantly up‐regulated, while *miR396b‐5p* was down‐regulated by Cd exposure (Fang *et al*., 2013). Thus, a complex mechanism may regulate the expression profiles of members of the same family.

A transcriptome‐wide analysis of the degradome was performed, and numerous target transcripts for the known and novel miRNAs were determined. For the miRNAs, 754 targets were identified by degradome sequencing (Table S6). Most targets of known miRNAs are conserved, including transcription factors, and those involved in signal transduction and plant response to heavy metals, all of which are found in other plant species (Cao *et al*., 2013; Zhang *et al*., 2012, 2014). Recent studies showed that a number of miRNAs regulate the expression of their targets through transcript cleavage or translation repression (Brodersen *et al*., 2008). An integrated analysis of miRNA expression profiles and their targets can help to identify the functional miRNA‐target modules involved in regulating specific biological processes (He *et al*., 2014; Pei *et al*., 2013). In this study, we identified 45 differentially expressed targets from 40 differentially expressed miRNAs by DEG analysis and RT‐qPCR (Table 3), and most of these miRNA‐target pairs showed reverse expression patterns (Figure 4). For example, *cme‐miR858_L‐1R+1* was significantly down‐regulated by Cd exposure, while its target *MYB12* (BMK. 57978) was significantly up‐regulated. However, *miR858* was also reported to be up‐regulated in roots exposed to different Pb concentrations in cotton (He *et al*., 2014). However, many miRNA‐target pairs involved in heavy metal stress in previous reports were not detected in our study, such as *miR604/LTP* (lipid transfer protein) (Huang *et al*., 2009) and *miR167/NRAMP* (Burklew *et al*., 2012; Srivastava *et al*., 2013). *S. alfredii* may have evolved some unique miRNA‐target regulatory mechanisms in response to Cd stress.

It was revealed that two hub genes, *ARF4* and *AAP3*, might play central roles in the regulation of Cd‐responsive genes by a coexpression regulatory network construction. Amino acid transport is highly regulated by environmental signals, such as light, cold, high salt and drought, and mechanical signals (Liu and Bush, 2006). In *Populus*, the expression levels of most AAP genes were down‐regulated by salt stress (Wu *et al*., 2015). Additionally, three amino acid transporters showed down‐regulated expression patterns in *S. nigrum* roots with a Cd treatment (Xu *et al*., 2012). Similarly, our investigation indicated that the expression of *AAP3* in *S. alfredii* exhibited a down‐regulated pattern under Cd stress (Figure 5i). Furthermore, many ARF transcription factors, as critical components of auxin signalling, play important roles in regulating various abiotic stressors (Jain and Khurana, 2009; Wang *et al*., 2010). The expression levels of numerous ARF genes change when plants respond to abiotic stresses in soya bean (Ha *et al*., 2013), *Sorghum bicolor* (Sekhwal *et al*., 2015) and banana (Hu *et al*., 2015). In this study, we found that the expression of *ARF4* was up‐regulated by Cd stress (Figure 5j). Thus, *AAP3* and *ARF4* are potentially relevant to Cd responses in *S. alfredii*.

In conclusion, our study reported an integrated analysis of the transcriptomics, sRNAs and degradome to generate a comprehensive resource focused on identifying key regulatory miRNA‐target circuits under Cd stress. A total of 87 721 unigenes and 356 miRNAs were identified, and 79 miRNAs were significantly differentially expressed and identified as Cd‐responsive miRNAs. The target genes for Cd‐responsive miRNAs functioned in signal transduction, response to heavy metal stress and secondary metabolite pathways. Furthermore, 45 differentially expressed targets were screened from 40 differentially expressed miRNAs using an integrated analysis of miRNA‐target expression profiles. Ninety‐one coexpressed genes formed a coregulation subnetwork in which two hub targets, *ARF4* and *AAP3*, might play a central role in controlling the transcriptomic regulation in response to Cd. These results will facilitate a comprehensive understanding of Cd hyperaccumulation in *S. alfredii*, and help elucidate miRNA‐mediated molecular mechanisms underlying plant responses to Cd stress and provide insights into Cd phytoremediation.

## Materials and methods

### Plant materials and Cd stress treatment

Hyperaccumulating *S. alfredii* plants were originally collected from a disused zinc/lead mining area in Quzhou City, Zhejiang Province, P. R. China, and then grown in an artificial climate chamber under control conditions (25–28°C, 16 h photoperiod). After 20 days in water culture, plants with similar growth status were treated with 400 μm CdCl_2_. Their roots, stems and leaves were sampled at 0 h (S1), 0.5 h (S2), 6 h (S3), 12 h (S4), 24 h (S5), 48 h (S6), 72 h (S7) and 96 h (S8), respectively.

All of the samples were frozen in liquid nitrogen immediately, and stored at –80°C. Total RNA was extracted using the Total RNA Purification Kit (NORGEN, Thorold, Canada). All RNA samples were treated with DNase I (TaKaRa, Dalian, China) to avoid genomic DNA contamination. RNA quality and purity were checked using denaturing 1.0% (p/v) agarose gel electrophoresis and a NanoDrop 2000 spectrophotometer (Thermo, Wilmington, DE) at 260/280 nm (ratio >2.0). The total mixed RNA samples, consisting of roots, stems and leaves, treated with 400 μM CdCl_2_ for eight different durations, were prepared for *de novo* transcriptome and degradome sequencing. Then, equal parts of RNA roots, stem and leaf samples for the same time point were mixed together for sRNA sequencing and DEG analysis.

### Transcriptome sequencing and *de novo* assembly analysis

The sequencing library for transcriptome analysis was prepared according to Illumina's kit. mRNA with Poly (A) was purified from the total RNA, using oligo(dT) magnetic beads, and then fragmented with an RNA fragmentation kit. First‐strand cDNA was synthesized using six random hexamer primers and a template of short fragments. Second‐strand cDNA was synthesized using the buffer, dNTPs, RNase H and DNA polymerase I and then ligated to sequencing adapters. Following the isolation of suitable cDNA fragments by gel electrophoresis analysis, and PCR enrichment, the final products were loaded onto an Illumina HiSeq^™^ 2000 platform for transcriptome sequencing. Total reads were trimmed of low‐quality and adapter sequences using the Illumina Pipeline, and then the remaining high‐quality reads were assembled into nonredundant unigenes using Trinity (http://trinityrnaseq.github.io/). Unigenes were tentatively identified based on the best hits against known sequences in the database.

### sRNA sequencing and miRNAs identification

Equal amounts of RNA samples from roots, stems and leaves at each of the eight different Cd treatment durations were pooled to generate eight sRNA libraries. The raw RNA reads generated by NGS from the eight sRNA libraries were processed to remove 5' and 3' adapters, and contaminated and low‐quality sequences, as well as those smaller than 18 nt, through Illumina's Genome Analyzer Pipeline V1.5. Then, the filtered reads were subjected to a further filtration step, to remove the common RNA families (rRNA, tRNA and snRNA) with a proprietary pipeline script ACGT101‐miR v4.2 (LC Sciences, Houston, TX). Then, the remaining clean and unique reads were aligned against the latest miRBase database, version 20.0 (http://www.mirbase.org/), using the BLAST algorithm to identify known miRNAs. The stem‐loop hairpin structures were aligned to sequencing reads and mature miRNAs from miRBase using Bowtie (Langmead *et al*., 2009). Four groups of miRNAs were revealed through bioinformatics analysis of sRNA sequencing based on the classification method (Ambady *et al*., 2012). The reads distribution was checked to meet the principles for miRNA prediction and authentic miRNAs were regarded as described previously (Tarver *et al*., 2012; Taylor *et al*., 2014). All of the sequencing data set, including transcriptome sequencing and sRNA sequencing results, were deposited into the NCBI SRA database under accession number SRP058333.

### Degradome sequencing, target identification and analysis

Equal amounts of all 24 RNA samples were mixed together to generate one degradome library. Then, the eight sRNA mixed samples and one degradome mixed sample were sent to Hangzhou LC‐Bio Co., Ltd (Hangzhou, China) for cDNA library construction and Illumina Genome Analyzer GA‐I Sequencing (Illumina, San Diego, CA). The extracted sequencing reads generated by degradome sequencing having 20 and 21 nucleotides were used to identify potentially cleaved targets by the CleaveL and 3.0 pipeline. Then, the degradome reads were mapped to the hyperaccumulating *S. alfredii* transcriptome data. The targets selected were categorized as 0, 1, 2, 3 and 4 as in a previous study (Xu *et al*., 2013; Yang *et al*., 2013). Based on the signatures (and abundances) along the hyperaccumulating *S. alfredii* transcriptome data, t‐plots were built for the high‐efficiency analysis of the potential miRNA targets. Finally, all of the identified potential target genes were subjected to an NCBI search using the BLASTX algorithm and a GO analysis.

### Differentially expressed target gene analysis

To discover the expression profiles of the target genes, eight independent libraries were constructed from each of the RNA samples from eight different Cd treatment durations. For each library, all of the sequences were processed to filter out adaptor sequences and low‐quality sequences. Then, all of the clean tags were mapped to the assembled unigenes of the hyperaccumulating *S. *alfredii for annotation. The reads per kb per million reads method was used to calculate the gene expression level. Then, a rigorous algorithm method was performed to identify the differentially expressed genes between two samples. The false discovery rate method was used to determine the *P‐*value threshold in multiple tests and analyses. The significantly differentially expressed genes among all of the different samples were judged by the threshold as follows: *P*‐value <0.005, false discovery rate ≤0.001 and the absolute value of log2 ratio ≥1. The heatmap of the differentially expressed miRNAs and their corresponding targets was constructed using the ggplot2 package in R (version 3.1.3).

### RT‐qPCR validation

To validate the high‐throughput sequencing results, RT‐qPCR on miRNAs and their targets was performed on a 7300 Real‐Time PCR System (Bio‐Rad, Hercules, CA). We chose 12 miRNA‐target pairs, including 12 differentially expressed miRNAs and 20 targets, for the RT‐qPCR analysis. The RNA samples used for RT‐qPCR were the same as for the experiments mentioned above. The reverse transcription reactions for miRNAs and cDNA were carried out using the SYBR^®^ PrimeScript^™^ miRNA RT‐PCR Kit (TaKaRa, Dalian, China) and the Superscript III First‐Strand Synthesis system followed by RNase H treatment (Invitrogen, Carlsbad, CA), respectively. The specific miRNA forward primers were designed based on the sequences of the respective miRNAs, and the primers for the target genes were designed using the online Primer3 program (http://frodo.wi.mit.edu/primer3/). *Beta‐tubulin* (*TUB*) was selected as a reference gene (Sang *et al*., 2013). The gene names, sequences and the primers used for RT‐qPCR analysis are available in File S1. RT‐qPCR reactions for miRNAs and their targets were performed in 96‐well plates using a SYBR^®^ Premix Ex Taq^™^ Kit (TaKaRa, Dalian, China) and SYBR^®^ PrimeScript^™^ miRNA RT‐PCR Kit (TaKaRa, Dalian, China). The amplification procedure and further data analysis were performed according to a previous study (Han *et al*., 2012). Each PCR reaction was repeated three times independently.

### Coexpression network construction

Construction of the network was performed using WGCNA to identify modules of highly correlated genes based on the DEG data (Langfelder and Horvath, 2008; Zhang and Horvath, 2005). Expression values with more than four zeros for each gene were selected and log2 transformed before being processed through the WGCNA package (version 1.46) in R (version 3.1.3). An extensive overview of WGCNA explaining the analysis steps can be found on the webpage: http://labs.genetics.ucla.edu/horvath/CoexpressionNetwork/Rpackages/WGCNA/Tutorials/index.html (Langfelder and Horvath, 2008). For quality control, we performed a clustering and principal components analysis based on the gene expression levels of the DGE data. The correlation networks were produced using the WGCNA package with the default power of six (Venables *et al*., 2012). All of the other parameters were used with the default values. Eigengenes were calculated for each gene coexpression module to visualize the gene expression patterns. The coexpressed genes with strong interconnection were defined as hub genes. Subsequently, we selected the differentially expressed target genes from the hub genes to analyse their edges. Results of coexpression analysis were visualized in Cytoscape (version 3.2.1) as described by Saito *et al*. (2012).

## Supporting information


**Figure S1** Length distributions of the unique sRNAs.

**Figure S2** Numbers of identified miRNAs in known miRNA families in *Sedum alfredii*. Graphical representation of the different members of conserved miRNA families by sequencing and bioinformatics prediction. ‘Other’ represents 34 of the known miRNA families containing only one member.

**Figure S3** Five categories based on target cleavage positions.

**Figure S4** WGCNA coexpression modules based on DEG data.

**Table S1** Overview of sRNA sequencing.

**Table S2** The list of miRNAs identified through the bioinformatics pipeline.

**Table S3** Length distribution of unique miRNA.

**Table S4** Profiles of novel miRNAs originating from predicted RNA hairpins.

**Table S5** miRNA genes supported by miRBase and reads coverage.

**Table S6** The predicted targets of known and novel miRNAs.

**Table S7** A complete list of *ARF4* and *AAP3* module assignments.

**Table S8** The edge genes annotated in the coexpression subnetwork of *ARF4* and *AAP3*.

**File S1** miRNA and their targets names, sequences and primers for RT‐qPCR.
