# Supplementary figures and images for: Integration of small RNAs, degradome and transcriptome sequencing in hyperaccumulator Sedum alfredii uncovers a complex regulatory network and provides insights into cadmium phytoremediation
Source: Plant Biotechnol J. 2016 Jan 23;14(6):1470–83. doi: 10.1111/pbi.12512 (PMC5066797; doi:10.1111/pbi.12512)

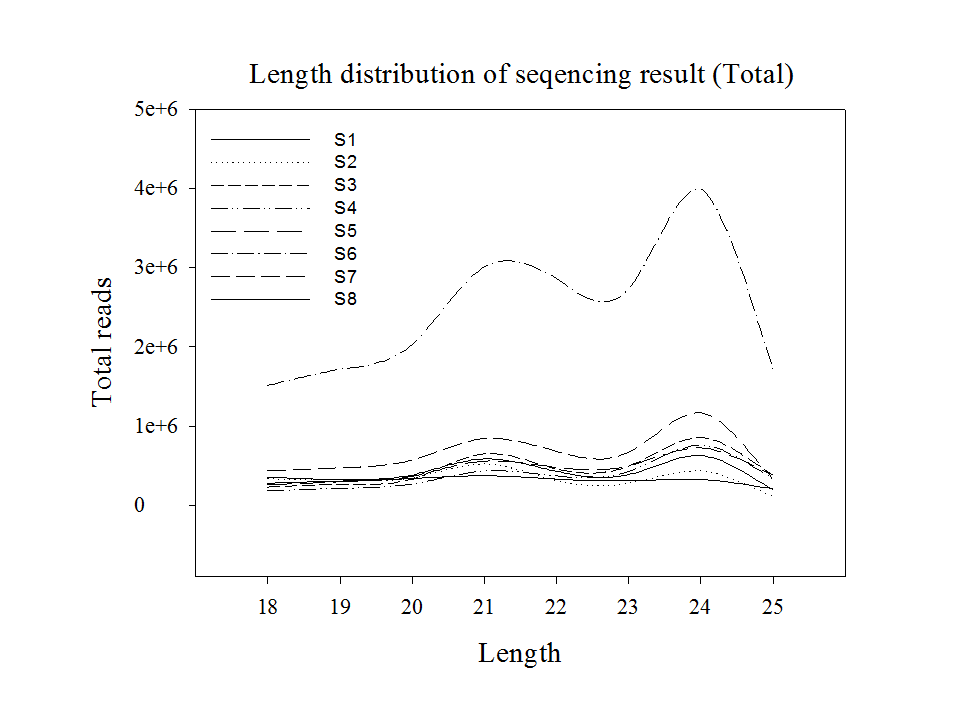

Supplement: Supplementary file 1 — Figure S1 Length distributions of the unique sRNAs. [file PBI-14-1470-s006.tif]

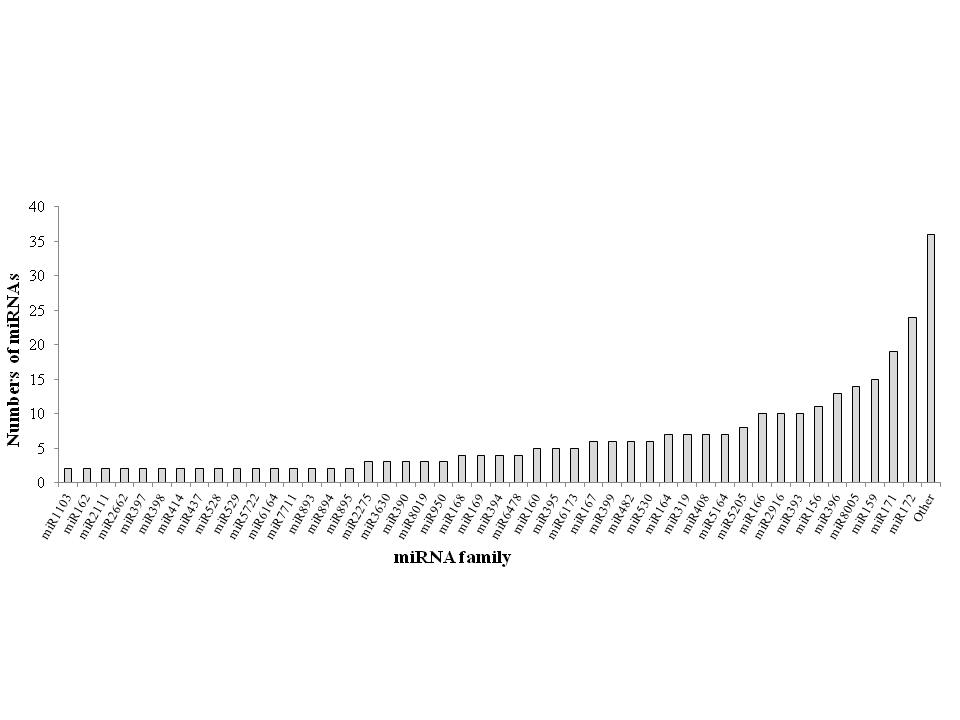

Supplement: Supplementary file 2 — Figure S2 Numbers of identified miRNAs in known miRNA families in Sedum alfredii. Graphical representation of the different members of conserved miRNA families by sequencing and bioinformatics prediction. ‘Other’ represents 34 of the known miRNA families containing only one member. [file PBI-14-1470-s011.tif]

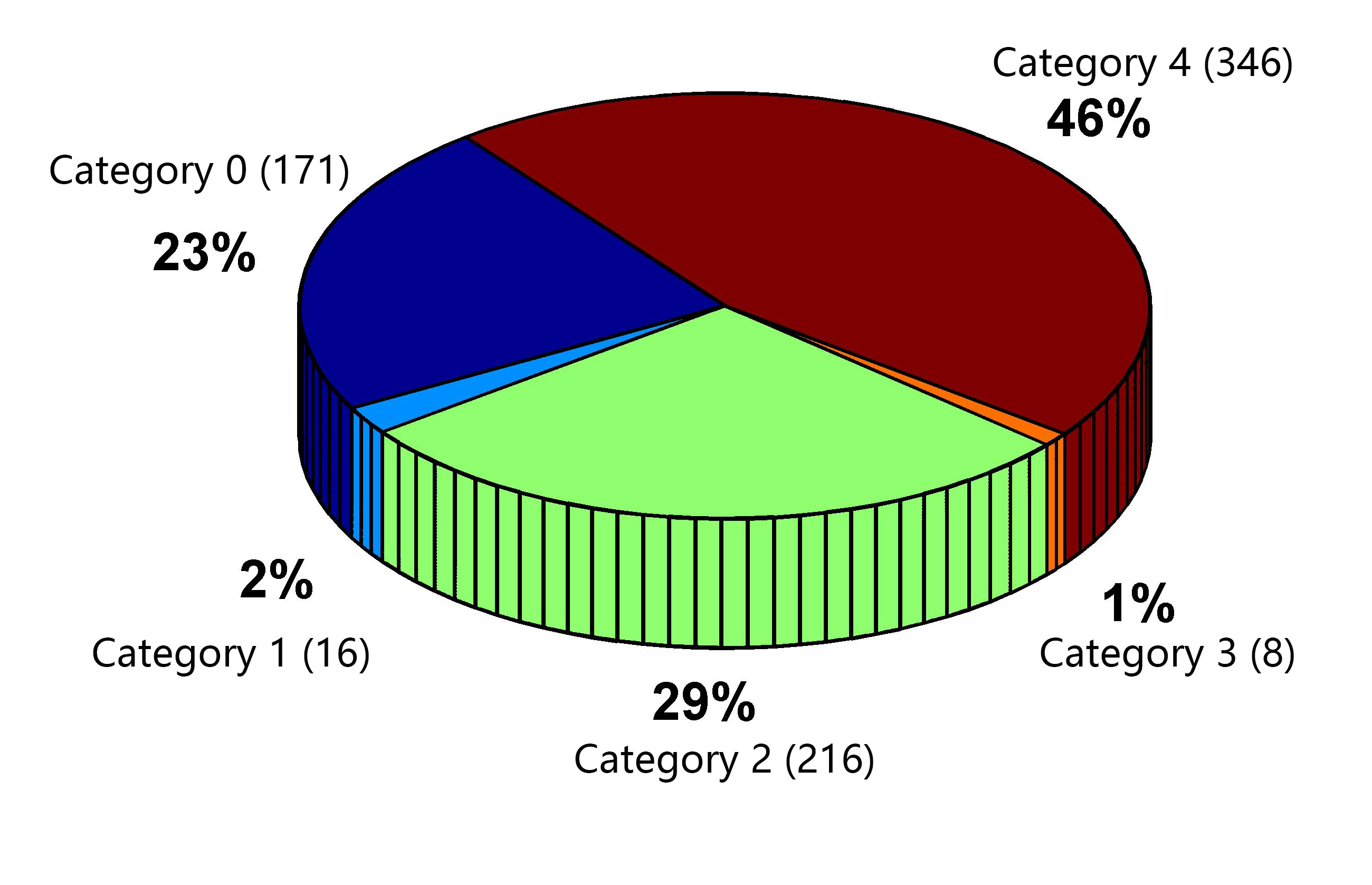

Supplement: Supplementary file 3 — Figure S3 Five categories based on target cleavage positions. [file PBI-14-1470-s010.jpg]

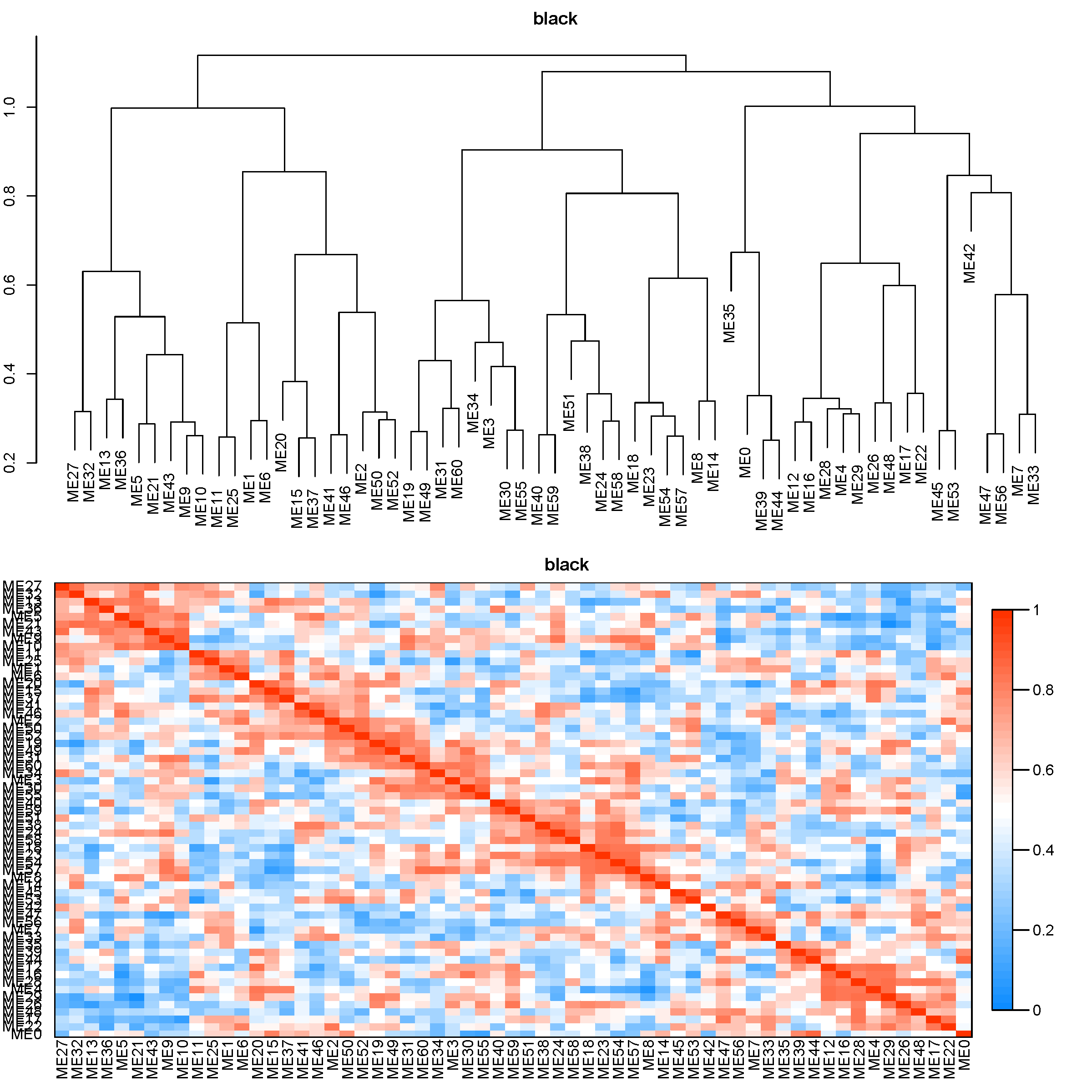

Supplement: Supplementary file 4 — Figure S4 WGCNA coexpression modules based on DEG data. [file PBI-14-1470-s012.tiff]
