# Supplementary material for: Integration of small RNAs, degradome and transcriptome sequencing in hyperaccumulator Sedum alfredii uncovers a complex regulatory network and provides insights into cadmium phytoremediation
Source: Plant Biotechnol J. 2016 Jan 23;14(6):1470–83. doi: 10.1111/pbi.12512 (PMC5066797; doi:10.1111/pbi.12512)
Supplement: Supplementary file 5 — Table S1 Overview of sRNA sequencing. [file PBI-14-1470-s001.docx]

**Table S1 Overview of sRNA sequencing**

| **Category** | **Ck (S1)** | **0.5h-Cd (S2)** | **6h-Cd (S3)** | **12h-Cd (S4)** |
| --- | --- | --- | --- | --- |
| **Raw reads** | 10,650,372 | 6,869,372 | 9,038,478 | 7,911,306 |
| **Adapter and length filter** | 6,579,650 | 3,190,923 | 3,663,438 | 3,715,185 |
| **Junk reads** | 5,519 | 11,557 | 13,331 | 11,647 |
| **Mappable reads** | 4,065,203 | 3,666,892 | 5,361,709 | 4,184,474 |
| **Mapped Rate** | 38.2% | 53.4% | 59.3% | 52.9% |
| **Total Clean Reads** | 2,475,251 | 2,659,057 | 3,663,345 | 2,990,567 |
| **Unique Clean Reads** | 493,037 | 859,390 | 1,023,607 | 906,347 |
| **Category** | **24h-Cd (S5)** | **48h-Cd (S6)** | **72h-Cd (S7)** | **96h-Cd (S8)** |
| **Raw reads** | 10,279,519 | 42,679,958 | 11,637,581 | 7,495,501 |
| **Adapter and length filter** | 5,068,803 | 15,180,023 | 4,225,452 (36.31%) | 2,995,770 |
| **Junk reads** | 15,602 | 183,268 | 25,611 (0.22%) | 24,809 |
| **Mappable reads** | 5,195,114 | 27,316,667 | 7,386,518 | 4,474,922 |
| **Mapped Rate** | 50.5% | 64.0% | 63.5% | 59.7% |
| **Total Clean Reads** | 3,563,015 | 19,571,439 | 5,170,301 | 3,296,298 |
| **Unique Clean Reads** | 686,831 | 4,986,693 | 1,724,091 | 1,188,086 |
