# Supplementary material for: Integration of small RNAs, degradome and transcriptome sequencing in hyperaccumulator Sedum alfredii uncovers a complex regulatory network and provides insights into cadmium phytoremediation
Source: Plant Biotechnol J. 2016 Jan 23;14(6):1470–83. doi: 10.1111/pbi.12512 (PMC5066797; doi:10.1111/pbi.12512)
Supplement: Supplementary file 7 — Table S3 Length distribution of unique miRNA. [file PBI-14-1470-s003.docx]

**Table S2 Length distribution of unique miRNA**

| length | Known miRNA | | Novel miRNA | |
| --- | --- | --- | --- | --- |
|  | Number | % | Number | % |
| 18 | 59 | 19.73 | 3 | 5.26 |
| 19 | 37 | 12.37 | 0 | 0.00 |
| 20 | 36 | 12.04 | 2 | 3.51 |
| 21 | 124 | 41.47 | 17 | 29.82 |
| 22 | 32 | 10.70 | 6 | 10.53 |
| 23 | 6 | 2.01 | 1 | 1.75 |
| 24 | 5 | 1.67 | 27 | 47.37 |
| 25 | 0 | 0.00 | 1 | 1.75 |
| All | 299 | 100.00 | 57 | 100.00 |
