# Supplementary material for: Integration of small RNAs, degradome and transcriptome sequencing in hyperaccumulator Sedum alfredii uncovers a complex regulatory network and provides insights into cadmium phytoremediation
Source: Plant Biotechnol J. 2016 Jan 23;14(6):1470–83. doi: 10.1111/pbi.12512 (PMC5066797; doi:10.1111/pbi.12512)
Supplement: Supplementary file 8 — Table S4 Profiles of novel miRNAs originating from predicted RNA hairpins. [file PBI-14-1470-s004.docx]

Table S3 Profiles of novel miRNAs

| Novel miRNAs | Mature sequence (5' to 3') | Length | dG | MFEI |
| --- | --- | --- | --- | --- |
| PC-5p-218348_44 | AGTTTTGGGACAATTAAATTGAAT | 24 | -55 | 0.90 |
| PC-3p-768319_10 | AGTTTTGTGTTAGGGTTTTTGATA | 24 | -55 | 0.90 |
| PC-5p-123438_88 | AAAGTTTAATGAAGGAAATGA | 21 | -85.20 | 1.40 |
| PC-3p-927250_10 | TTCCCTTAAAATTTTCTTTCCTCT | 24 | -85.20 | 1.40 |
| PC-5p-51499_238 | TGTGGGAAAATTGAGAGGAAA | 21 | -82.90 | 1.60 |
| PC-3p-1871482_4 | AAGAAAAAGATCATTTCCTTCGTT | 24 | -82.90 | 1.60 |
| PC-5p-913758_8 | AAAGCTGTCATAGAGCAATGGCCT | 24 | -88.40 | 1.00 |
| PC-3p-1072007_7 | AGACTTAATGGAGTTTGCCTTGAC | 24 | -88.40 | 1.00 |
| PC-5p-207477_71 | TTGTTTAGTTCTCCTTTATT | 20 | -56 | 0.90 |
| PC-3p-858011_8 | TAGAGGGAAGTTGTGAGCGTT | 21 | -56 | 0.90 |
| PC-5p-895055_8 | AACTCGCAGGACAGCACCAGC | 21 | -71.40 | 1.00 |
| PC-3p-2783006_3 | AAAGGATTGGGATCCACTCAAAAC | 24 | -71.40 | 1.00 |
| PC-5p-860174_8 | GGTATGTTGTTTGGCCCGAGG | 21 | -57.60 | 0.90 |
| PC-3p-3238323_4 | TCGGACCAGGTTTCATTCCTG | 21 | -57.60 | 0.90 |
| PC-5p-683698_12 | GATTCGACGGTTTCTGCAATC | 21 | -46.30 | 1.10 |
| PC-3p-49685_244 | TTGCAAAAGCCGTCCCAATCAC | 22 | -46.30 | 1.10 |
| PC-5p-126214_87 | ATTTCCTTCATTAAACTTTCC | 21 | -90.30 | 1.20 |
| PC-3p-343059_26 | TCCACTACTTTTTCCCGCATC | 21 | -90.30 | 1.20 |
| PC-5p-113562_98 | CTCGGAGTTGGACTGAAACCC | 21 | -140.10 | 1.50 |
| PC-3p-1735947_5 | CCGACGACGAATGATGCGAGCT | 22 | -140.10 | 1.50 |
| PC-5p-548039_17 | TCGTTGAGGACCGTCTCATGGAGA | 24 | -97.70 | 0.90 |
| PC-3p-1503821_5 | ATCTCGCGAGATCCGTCTAATGGA | 24 | -97.70 | 0.90 |
| PC-5p-98092_120 | TATCGATCGAAATCGAAGCCC | 21 | -51.20 | 1.10 |
| PC-3p-3544953_2 | TTTCGAATCGATAGGATTAAGG | 22 | -51.20 | 1.10 |
| PC-5p-3414764_2 | AAACGGCCGGAAACAGCGTTGACC | 24 | -112.80 | 1.10 |
| PC-3p-4452358_2 | AACGCCGTTTCCCAGCCGTTTCCC | 24 | -112.80 | 1.10 |
| PC-5p-865443_8 | GATTCGATGGTTTTTGCAATC | 21 | -43.30 | 1.10 |
| PC-3p-1662_4367 | CTGCAGAAGCCATCGAAATCGC | 22 | -43.30 | 1.10 |
| PC-5p-2679901_5 | TGCGGGAAAATTGAGAGGAAA | 21 | -56.20 | 1.40 |
| PC-3p-1507684_5 | CTTAAAATTTTCTTTCCTCTACT | 23 | -56.20 | 1.40 |
| PC-5p-308784_31 | ATTGAGAAACGTTTAATGAAGAAA | 24 | -56 | 1.10 |
| PC-3p-375067_23 | AGGAAAGTTTAATGAAGAAAAAGA | 24 | -56 | 1.10 |
| PC-5p-1037697_8 | TTGGAACGGAATGAAATGATGAAT | 24 | -49.80 | 1.30 |
| PC-3p-2078237_4 | ATCCTTACATTCCATTCCAACGAA | 24 | -49.80 | 1.30 |
| PC-5p-1043025_7 | AAAAGTAGAGGAAAGAAAATTTTA | 24 | -59.60 | 1.20 |
| PC-3p-3461203_2 | TATGTAGTTTTGGGTGAC | 18 | -49.30 | 1.10 |
| PC-3p-1057448_8 | ATTTACCGTTTTACCGTTTGCTCT | 24 | -28.90 | 1.20 |
| PC-5p-462776_18 | ATGGAAGGATGAATATGGATTGAT | 24 | -26 | 0.90 |
| PC-5p-754180_10 | AAGGGAAAGTTTAATGAATGAAAT | 24 | -52.80 | 1.00 |
| PC-5p-2007215_3 | TAATGTTGCGACCACTTGGAAGGA | 24 | -68.60 | 1.20 |
| PC-3p-803056_10 | AATCACTCAAGAATTCAATCATAT | 24 | -52.50 | 1.10 |
| PC-5p-322854_27 | ACTTGGAAGGAATGAAATGACAAT | 24 | -66.30 | 0.90 |
| PC-5p-3760_2402 | TTTTCTTGACCTTGTAAGACC | 21 | -60 | 1.30 |
| PC-5p-108723_104 | AAGGGAAAGTTTAATGAAGAAAAA | 24 | -50.80 | 1.30 |
| PC-5p-11769136_1 | GATTGGATTACTAAGAATTAACTCA | 25 | -21.20 | 0.90 |
| PC-3p-976855_7 | GGTTCTGAGTAAACAATGAAACAT | 24 | -48.10 | 1.00 |
| PC-5p-713544_11 | GCGGGGAAACGGCTGGGAAACGGC | 24 | -147.10 | 1.20 |
| PC-3p-1468858_5 | ATTCCAACGAAGTGGTCGCAACAT | 24 | -57.20 | 1.50 |
| PC-3p-2242608_4 | TTTTTACCGATTGCTCTCCCCTAC | 24 | -57.90 | 1.20 |
| PC-3p-125533_95 | TTGCACTGACCGTCGTAGTCGC | 22 | -47.10 | 1.00 |
| PC-3p-1650016_5 | CGAAAGAGAATCTGGACA | 18 | -27.40 | 0.90 |
| PC-5p-222566_46 | AGCTGCTTTTATATGGATCCC | 21 | -63.80 | 0.90 |
| PC-5p-905043_8 | TGTGGGAAAATTGGGAGGAAA | 21 | -52.60 | 1.40 |
| PC-3p-4440015_2 | GCATTGTGGAGTTTCTGGATCA | 22 | -27 | 0.90 |
| PC-5p-20273079_1 | CAAATCTGAGGTTCTGAA | 18 | -28.90 | 0.90 |
| PC-3p-1637833_4 | TTGGACTGAAACCCTAAAGCC | 21 | -143.10 | 1.20 |
| PC-5p-6279784_1 | AATAGATTTTAGCCAGTGAA | 20 | -23.90 | 0.90 |
| Average |  | 22.44 | -65.62 | 1.12 |
