# Supplementary material for: Integration of small RNAs, degradome and transcriptome sequencing in hyperaccumulator Sedum alfredii uncovers a complex regulatory network and provides insights into cadmium phytoremediation
Source: Plant Biotechnol J. 2016 Jan 23;14(6):1470–83. doi: 10.1111/pbi.12512 (PMC5066797; doi:10.1111/pbi.12512)
Supplement: Supplementary file 9 — Table S5 miRNA genes supported by miRBase and reads coverage. [file PBI-14-1470-s005.docx]

**Table S4. The list of miRNAs with abundant expression levels and displayed canonical hairpin structures.**

| Pre-miRNA Index | miRNA_Index | miR_name | miR_seq | len | genomeID | #mir | group | hairpinLen | Expression level |
| --- | --- | --- | --- | --- | --- | --- | --- | --- | --- |
| 1 | 1 | mtr-miR160a | TGCCTGGCTCCCTGTATGCCA | 21 | Sedum_Unigene_BMK.49523 | 55 | gp2a | 121 | middle |
| 2 | 3 | mtr-miR164a_1ss17GA | TGGAGAAGCAGGGCACATGCA | 21 | Sedum_Unigene_BMK.49242 | 50 | gp2a | 69 | middle |
| 3 | 6 | mtr-miR166a | TCGGACCAGGCTTCATTCCCC | 21 | Sedum_Unigene_BMK.49232 | 120 | gp2a | 141 | high |
| 4 | 8 | mtr-miR166c_2ss20TC21CA | TCGGACCAGGCTTCATTCCCA | 21 | Sedum_Unigene_BMK.53452 | 123 | gp2a | 94 | middle |
| 5 | 9 | mtr-miR168b | TCGCTTGGTGCAGGTCGGGAA | 21 | Sedum_Unigene_BMK.54763 | 26 | gp2a | 149 | high |
| 6 | 12 | mtr-miR171c_2ss12TC21TC | TGATTGAGCCGCGCCAATATC | 21 | Sedum_Unigene_BMK.12439 | 40 | gp2a | 127 | middle |
| 7 | 14 | csi-miR171a_R+1 | TTGAGCCGCGCCAATATCACT | 21 | Sedum_Unigene_BMK.22730 | 46 | gp2a | 175 | middle |
| 8 | 16 | csi-miR171a_R+2_1ss9CT | TTGAGCCGTGCCAATATCACGT | 22 | Sedum_Unigene_BMK.58017 | 72 | gp2a | 89 | middle |
| 9 | 17 | mes-MIR172c-p5_1ss12AG | CAGCATCATCAGGATTCTCAC | 21 | Sedum_Unigene_BMK.50491 | 6 | gp2a | 99 | low |
| 9 | 18 | stu-MIR172d-p3_1ss5GT | TGTGTGAATCTTGATGATG | 19 | Sedum_Unigene_BMK.50491 | 6 | gp2a | 99 | low |
| 10 | 20 | mtr-miR172d-3p_R+1 | AGAATCTTGATGATGCTGCAT | 21 | Sedum_Unigene_BMK.50491 | 69 | gp2a | 135 | middle |
| 12 | 22 | mtr-miR393a | TCCAAAGGGATCGCATTGATC | 21 | Sedum_Unigene_BMK.39909 | 20 | gp2a | 106 | middle |
| 15 | 27 | mtr-MIR2609a-p5_1ss17AT | TTGGATGATGATCAGATG | 18 | Sedum_Unigene_BMK.25517 | 2 | gp2a | 61 | middle |
| 1 | 1 | mtr-miR156b-5p_1ss20CT | TGACAGAAGAGAGTGAGCAT | 20 | Sedum_Unigene_BMK.11432 | 86 | gp2b | 136 | low |
| 4 | 4 | mtr-miR156b-5p_R+1_1ss14TG | TGACAGAAGAGAGGGAGCACA | 21 | Sedum_Unigene_BMK.31642 | 84 | gp2b | 133 | low |
| 5 | 5 | mtr-miR156a_L+1R-1_1ss12GT | TTGACAGAAGATAGAGAGCAC | 21 | Sedum_Unigene_BMK.55559 | 133 | gp2b | 50 | middle |
| 6 | 6 | mtr-miR156e_R-2 | TTGACAGAAGATAGAGAGC | 19 | Sedum_Unigene_BMK.58069 | 61 | gp2b | 73 | low |
| 7 | 7 | mtr-miR156b-5p_1ss15GA | TGACAGAAGAGAGTAAGCAC | 20 | Sedum_Unigene_BMK.58705 | 79 | gp2b | 120 | low |
| 9 | 8 | mtr-miR159a | TTTGGATTGAAGGGAGCTCTA | 21 | Sedum_Unigene_BMK.57127 | 41 | gp2b | 53 | high |
| 10 | 9 | pde-miR159_R-1 | TTTGGTTTGAAGGGAGCTCT | 20 | Sedum_Unigene_BMK.1477 | 8 | gp2b | 150 | low |
| 16 | 15 | mtr-miR160a_1ss21AT | TGCCTGGCTCCCTGTATGCCT | 21 | Sedum_Unigene_BMK.25046 | 68 | gp2b | 81 | low |
| 17 | 15 | mtr-miR160a_1ss21AT | TGCCTGGCTCCCTGTATGCCT | 21 | Sedum_Unigene_BMK.61725 | 63 | gp2b | 62 | low |
| 18 | 16 | mtr-miR160c | TGCCTGGCTCCCTGAATGCCA | 21 | Sedum_Unigene_BMK.37248 | 15 | gp2b | 115 | low |
| 20 | 19 | mtr-miR164a | TGGAGAAGCAGGGCACGTGCA | 21 | Sedum_Unigene_BMK.10628 | 50 | gp2b | 141 | middle |
| 25 | 24 | mtr-miR166a_L+3 | ATCTCGGACCAGGCTTCATTCCCC | 24 | Sedum_Unigene_BMK.82962 | 123 | gp2b | 129 | middle |
| 30 | 29 | mtr-miR169a_1ss21AG | CAGCCAAGGATGACTTGCCGG | 21 | Sedum_Unigene_BMK.49897 | 56 | gp2b | 147 | middle |
| 33 | 33 | mtr-miR171d_2ss12TC13GA | TGATTGAGCCGCACCAATATC | 21 | Sedum_Unigene_BMK.55803 | 22 | gp2b | 103 | middle |
| 39 | 40 | ptc-MIR172a-p5_1ss1GT | TCAGCATCATCAAGATTCACA | 21 | Sedum_Unigene_BMK.22776 | 7 | gp2b | 140 | low |
| 45 | 45 | mtr-miR172b_1ss1AG | GGAATCTTGATGATGCTGCAT | 21 | Sedum_Unigene_BMK.50884 | 66 | gp2b | 127 | middle |
| 46 | 46 | ptc-MIR172a-p5 | GCAGCATCATCAAGATTCACA | 21 | Sedum_Unigene_BMK.50884 | 52 | gp2b | 130 | middle |
| 52 | 51 | mtr-miR172a | AGAATCCTGATGATGCTGCAG | 21 | Sedum_Unigene_BMK.61399 | 18 | gp2b | 79 | low |
| 54 | 54 | mtr-miR319a-3p_R+1_1ss15GT | TTGGACTGAAGGGATCTCCCT | 21 | Sedum_Unigene_BMK.56060 | 58 | gp2b | 76 | high |
| 57 | 56 | mtr-miR393a_R+1_1ss21CT | TCCAAAGGGATCGCATTGATTC | 22 | Sedum_Unigene_BMK.62158 | 20 | gp2b | 119 | low |
| 58 | 58 | mtr-miR393a_R-3_1ss18GT | TCCAAAGGGATCGCATTT | 18 | Sedum_Unigene_BMK.48704 | 19 | gp2b | 131 | low |
| 60 | 59 | mtr-miR393a_R-3_1ss18GA | TCCAAAGGGATCGCATTA | 18 | Sedum_Unigene_BMK.48705 | 19 | gp2b | 132 | low |
| 64 | 63 | csi-miR394_L+1 | TTTGGCATTCTGTCCACCTCC | 21 | Sedum_Unigene_BMK.60766 | 18 | gp2b | 100 | middle |
| 65 | 64 | mtr-miR395a_1ss1AC | CTGAAGTGTTTGGGGGAACTC | 21 | Sedum_Unigene_BMK.40110 | 86 | gp2b | 102 | middle |
| 66 | 64 | mtr-miR395a_1ss1AC | CTGAAGTGTTTGGGGGAACTC | 21 | Sedum_Unigene_BMK.79413 | 86 | gp2b | 134 | middle |
| 67 | 65 | mtr-miR395a_L-1_1ss21CG | TGAAGTGTTTGGGGGAACTG | 20 | Sedum_Unigene_BMK.43494 | 78 | gp2b | 99 | low |
| 70 | 69 | ata-miR396a-5p_R-1 | TCCACAGGCTTTCTTGAACT | 20 | Sedum_Unigene_BMK.1449 | 5 | gp2b | 118 | middle |
| 71 | 69 | ata-miR396a-5p_R-1 | TCCACAGGCTTTCTTGAACT | 20 | Sedum_Unigene_BMK.48500 | 5 | gp2b | 135 | middle |
| 73 | 71 | ata-miR396a-5p_1ss21GA | TCCACAGGCTTTCTTGAACTA | 21 | Sedum_Unigene_BMK.32677 | 5 | gp2b | 129 | low |
| 74 | 72 | mtr-miR396b-5p | TTCCACAGCTTTCTTGAACTG | 21 | Sedum_Unigene_BMK.51907 | 55 | gp2b | 136 | middle |
| 75 | 73 | mtr-miR396b-5p_L-3 | CACAGCTTTCTTGAACTG | 18 | Sedum_Unigene_BMK.51907 | 23 | gp2b | 136 | low |
| 76 | 74 | ata-miR396a-5p | TCCACAGGCTTTCTTGAACTG | 21 | Sedum_Unigene_BMK.53890 | 5 | gp2b | 82 | middle |
| 77 | 75 | mtr-miR396a-5p_1ss21TG | TTCCACAGCTTTCTTGAACTG | 21 | Sedum_Unigene_BMK.54058 | 57 | gp2b | 53 | middle |
| 78 | 76 | mtr-miR396a-5p_1ss18AG | TTCCACAGCTTTCTTGAGCTT | 21 | Sedum_Unigene_BMK.54058 | 29 | gp2b | 79 | low |
| 80 | 78 | cme-miR399a_L+2R-2 | ACTGCCAAAGGAGATTTGCCC | 21 | Sedum_Unigene_BMK.46167 | 17 | gp2b | 83 | low |
| 81 | 79 | mtr-miR399l_1ss1TC | CGCCAAAGGAGAGTTGCCCTG | 21 | Sedum_Unigene_BMK.50636 | 36 | gp2b | 121 | middle |
| 82 | 80 | mtr-miR399c_1ss13TG | TGCCAAAGGAGAGTTGCCCTG | 21 | Sedum_Unigene_BMK.8460 | 40 | gp2b | 157 | low |
| 83 | 81 | mtr-miR408-3p_L-1R-2_1ss19GC | TGCACTGCCTCTTCCCTC | 18 | Sedum_Unigene_BMK.49832 | 26 | gp2b | 63 | low |
| 84 | 81 | mtr-miR408-3p_L-1R-2_1ss19GC | TGCACTGCCTCTTCCCTC | 18 | Sedum_Unigene_BMK.59701 | 26 | gp2b | 74 | low |
| 102 | 95 | smo-MIR1103-p5_1ss17TC | CTTAGGATAGTTGGAGCT | 18 | Sedum_Unigene_BMK.57121 | 6 | gp2b | 105 | low |
| 102 | 96 | ccl-MIR171-p3_1ss4GC | TTTCATTGAGCCGCGCCA | 18 | Sedum_Unigene_BMK.57121 | 6 | gp2b | 105 | low |
| 108 | 101 | mtr-MIR2592bj-p3_1ss12TC | ATTCCCACTGTCCCTGTC | 18 | Sedum_Unigene_BMK.57121 | 6 | gp2b | 121 | middle |
| 115 | 107 | peu-MIR2916-p5_1ss15TG | CTCGAAGACGATCAGATA | 18 | Sedum_Unigene_BMK.55105 | 1 | gp2b | 72 | middle |
| 118 | 110 | han-miR3630-3p_L-2R-1_1ss21TA | TGGGAATCTCTCTGATGCA | 19 | Sedum_Unigene_BMK.82935 | 1 | gp2b | 94 | middle |
| 119 | 110 | han-miR3630-3p_L-2R-1_1ss21TA | TGGGAATCTCTCTGATGCA | 19 | Sedum_Unigene_BMK.85559 | 1 | gp2b | 94 | middle |
| 141 | 125 | hbr-MIR6173-p3_1ss1GC | CGAATGGGATTAGATACC | 18 | Sedum_Unigene_BMK.16133 | 1 | gp2b | 65 | middle |
| 147 | 132 | hbr-MIR6485-p5_1ss4AG | TTCGGCAGATTTGGATTCCTATA | 23 | Sedum_Unigene_BMK.46883 | 1 | gp2b | 74 | middle |
| 159 | 142 | stu-MIR8005c-p5_1ss13AG | TTAGGGTTTAGGGTTTAGGGT | 21 | Sedum_Unigene_BMK.60743 | 2 | gp2b | 72 | middle |
| 161 | 144 | stu-MIR8005c-p3_1ss12AG | TAGGGTTTAGGGTTTAGGGTTT | 22 | Sedum_Unigene_BMK.66139 | 2 | gp2b | 55 | middle |
| 162 | 145 | stu-MIR8005a-p3_1ss21GT | TTAGGGTTTAGGGTTTAGGGT | 21 | Sedum_Unigene_BMK.84630 | 2 | gp2b | 120 | middle |
| 1 | 1 | ptc-miR156a_R+1_1ss15GA | TGACAGAAGAGAGTAAGCACT | 21 |  | 84 | gp3 | 92 | middle |
| 1 | 2 | bra-miR156e-3p_3ss8CA15T-23TA | TGCTCACATCTCTTCTGTCAGA | 22 |  | 84 | gp3 | 92 | middle |
| 2 | 3 | nta-miR156g_L+1R+1 | TTGACAGAAGATAGAGAGCACT | 22 |  | 42 | gp3 | 112 | middle |
| 2 | 4 | mtr-miR156h-3p_3ss6TC8AG10TG | GCTCTCTGTGCTTCTGTCATC | 21 |  | 42 | gp3 | 112 | middle |
| 4 | 6 | far-miR159_R+1_1ss21GC | TTTGGATTGAAGGGAGCTCTCT | 22 |  | 24 | gp3 | 195 | middle |
| 5 | 7 | sof-miR159c_1ss1CG | GTTGGATTGAAGGGAGCTCCT | 21 |  | 4 | gp3 | 172 | middle |
| 6 | 8 | bra-miR162-5p_L+1 | TGGAGGCAGCGGTTCATCGATC | 22 |  | 19 | gp3 | 120 | middle |
| 6 | 9 | bra-miR162-3p_1ss8AG | TCGATAAGCCTCTGCATCCAG | 21 |  | 19 | gp3 | 120 | middle |
| 7 | 10 | bra-miR164b-5p_1ss20CT | TGGAGAAGCAGGGCACGTGTG | 21 |  | 51 | gp3 | 106 | middle |
| 9 | 12 | cpa-miR167d_R-1 | TGAAGCTGCCAGCATGATCTG | 21 |  | 76 | gp3 | 78 | middle |
| 9 | 13 | ptc-miR167f-3p_2ss9TC20CT | AGATCATGCGGCAGTTTCATC | 21 |  | 76 | gp3 | 78 | middle |
| 11 | 15 | sof-miR168a | TCGCTTGGTGCAGATCGGGAC | 21 |  | 27 | gp3 | 99 | middle |
| 11 | 16 | mtr-miR168c-3p_2ss12TC16CG | CCCGCCTTGCACCAAGTGAAT | 21 |  | 27 | gp3 | 99 | middle |
| 12 | 17 | stu-miR171a-5p | TATTGGCCTGGTTCACTCAGA | 21 |  | 47 | gp3 | 103 | low |
| 12 | 35 | mtr-miR171c_L-3R+1 | TTGAGCCGTGCCAATATTT | 19 |  | 47 | gp3 | 103 | low |
| 14 | 18 | sly-miR171d_R+2 | TTGAGCCGCGCCAATATCACGC | 22 |  | 25 | gp3 | 121 | low |
| 17 | 21 | mtr-miR172c-5p_L+1R-1 | TGTAGCATCATCAAGATTCAC | 21 |  | 67 | gp3 | 167 | low |
| 18 | 23 | ata-miR319-3p_1ss9AG | ACTGGATGGCGCGGGAGCTAA | 21 |  | 1 | gp3 | 189 | middle |
| 20 | 25 | ata-miR390-5p | AAGCTCAGGAGGGATAGCGCC | 21 |  | 26 | gp3 | 138 | low |
| 20 | 26 | ata-miR390-3p | CGCTATCTATCCTGAGCTCC | 20 |  | 26 | gp3 | 138 | low |
| 21 | 27 | bra-miR390-5p | AAGCTCAGGAGGGATAGCGCC | 21 |  | 6 | gp3 | 105 | low |
| 22 | 28 | bdi-miR393a_R+1_1ss21CT | TCCAAAGGGATCGCATTGATTT | 22 |  | 20 | gp3 | 131 | middle |
| 24 | 30 | rco-miR395a_R+2_1ss21CT | CTGAAGTGTTTGGGGGAACTTTT | 23 |  | 85 | gp3 | 68 | low |
| 25 | 31 | cme-miR396e_1ss1TG | GTCCACGGCTTTCTTGAACTG | 21 |  | 57 | gp3 | 170 | low |
| 25 | 32 | mtr-miR396b-3p | GTTCAATAAAGCTGTGGGAAG | 21 |  | 57 | gp3 | 170 | middle |
| 28 | 35 | bdi-miR398a_1ss1TC | CGTGTTCTCAGGTCGCCCCTG | 21 |  | 14 | gp3 | 138 | middle |
| 30 | 37 | mtr-miR399b | TGCCAAAGGAGAGCTGCCCTG | 21 |  | 30 | gp3 | 96 | low |
| 31 | 38 | stu-miR399a-3p | TGCCAAAGGAGAGCTGCCCTG | 21 |  | 14 | gp3 | 62 | low |
| 32 | 39 | bra-miR403-3p | TTAGATTCACGCACAAACTCG | 21 |  | 11 | gp3 | 107 | low |
| 33 | 40 | cme-miR408_L-1R+2 | TGCACTGCCTCTTCCCTGGCTG | 22 |  | 24 | gp3 | 125 | middle |
| 34 | 41 | smo-miR408 | TGCACTGCCTCTTCCCTGGCTG | 22 |  | 3 | gp3 | 107 | middle |
| 35 | 42 | ptc-miR408-3p | ATGCACTGCCTCTTCCCTGGC | 21 |  | 3 | gp3 | 104 | middle |
| 36 | 43 | bdi-miR444c | TGCAGTTGTTGTCTCAAGCTT | 21 |  | 5 | gp3 | 124 | low |
| 39 | 48 | bdi-miR529-5p | AGAAGAGAGAGAGTACAGCCT | 21 |  | 6 | gp3 | 107 | middle |
| 39 | 49 | bdi-miR529-3p | GCTGTACCCTCTCTCTTCTTC | 21 |  | 6 | gp3 | 107 | low |
| 40 | 50 | ptc-miR530a_R+1 | TGCATTTGCACCTGCACCTTT | 21 |  | 9 | gp3 | 144 | middle |
| 41 | 51 | ptc-miR530b_R+1_1ss17TC | TGCATTTGCACCTGCACCTTT | 21 |  | 2 | gp3 | 172 | middle |
| 43 | 53 | rco-miR535 | TGACAACGAGAGAGAGCACGC | 21 |  | 10 | gp3 | 89 | middle |
| 44 | 54 | bdi-miR827-3p | TTAGATGACCATCAGCAAACA | 21 |  | 6 | gp3 | 160 | middle |
| 46 | 56 | cme-miR858_L-1R+1 | CTCGTTGTCTGTTCGACCTTG | 21 |  | 1 | gp3 | 154 | middle |
| 47 | 57 | ppt-miR894_R+1_1ss11CA | CGTTTCACGTAGGGTTCACCA | 21 |  | 1 | gp3 | 59 | high |
| 56 | 66 | bra-miR2111a-5p | TAATCTGCATCCTGAGGTTTA | 21 |  | 23 | gp3 | 161 | middle |
| 63 | 74 | bdi-miR5054_1ss10TA | TCCCCACGGACGGCGCCA | 18 |  | 1 | gp3 | 96 | middle |
| 64 | 75 | rgl-miR5139_L-1 | AACCTGGCTCTGATACCA | 18 |  | 2 | gp3 | 144 | high |
| 70 | 81 | ptc-miR6478_1ss21GA | CCGACCTTAGCTCAGTTGGTA | 21 |  | 1 | gp3 | 138 | middle |
| 1 | 1 | PC-5p-218348_44 | AGTTTTGGGACAATTAAATTGAAT | 24 | Sedum_Unigene_BMK.60619 | 0 | gp4 | 184 | middle |
| 2 | 3 | PC-5p-123438_88 | AAAGTTTAATGAAGGAAATGA | 21 | Sedum_Unigene_BMK.61478 | 0 | gp4 | 136 | middle |
| 2 | 4 | PC-3p-927250_10 | TTCCCTTAAAATTTTCTTTCCTCT | 24 | Sedum_Unigene_BMK.61478 | 0 | gp4 | 136 | middle |
| 3 | 5 | PC-5p-51499_238 | TGTGGGAAAATTGAGAGGAAA | 21 | Sedum_Unigene_BMK.61478 | 0 | gp4 | 136 | middle |
| 5 | 9 | PC-5p-207477_71 | TTGTTTAGTTCTCCTTTATT | 20 | Sedum_Unigene_BMK.60676 | 0 | gp4 | 64 | middle |
| 8 | 16 | PC-3p-49685_244 | TTGCAAAAGCCGTCCCAATCAC | 22 | Sedum_Unigene_BMK.41923 | 0 | gp4 | 95 | middle |
| 9 | 17 | PC-5p-126214_87 | ATTTCCTTCATTAAACTTTCC | 21 | Sedum_Unigene_BMK.61478 | 0 | gp4 | 136 | middle |
| 9 | 18 | PC-3p-343059_26 | TCCACTACTTTTTCCCGCATC | 21 | Sedum_Unigene_BMK.61478 | 0 | gp4 | 136 | middle |
| 10 | 19 | PC-5p-113562_98 | CTCGGAGTTGGACTGAAACCC | 21 | Sedum_Unigene_BMK.62288 | 0 | gp4 | 190 | middle |
| 11 | 21 | PC-5p-548039_17 | TCGTTGAGGACCGTCTCATGGAGA | 24 | Sedum_Unigene_BMK.58685 | 0 | gp4 | 108 | middle |
| 12 | 23 | PC-5p-98092_120 | TATCGATCGAAATCGAAGCCC | 21 | Sedum_Unigene_BMK.46948 | 0 | gp4 | 95 | middle |
| 14 | 28 | PC-3p-1662_4367 | CTGCAGAAGCCATCGAAATCGC | 22 | Sedum_Unigene_BMK.47281 | 0 | gp4 | 107 | middle |
| 16 | 31 | PC-5p-308784_31 | ATTGAGAAACGTTTAATGAAGAAA | 24 | Sedum_Unigene_BMK.49720 | 0 | gp4 | 131 | middle |
| 16 | 32 | PC-3p-375067_23 | AGGAAAGTTTAATGAAGAAAAAGA | 24 | Sedum_Unigene_BMK.49720 | 0 | gp4 | 131 | middle |
| 25 | 42 | PC-5p-322854_27 | ACTTGGAAGGAATGAAATGACAAT | 24 | Sedum_Unigene_BMK.22232 | 0 | gp4 | 182 | middle |
| 26 | 43 | PC-5p-3760_2402 | TTTTCTTGACCTTGTAAGACC | 21 | Sedum_Unigene_BMK.49414 | 0 | gp4 | 143 | middle |
| 27 | 44 | PC-5p-108723_104 | AAGGGAAAGTTTAATGAAGAAAAA | 24 | Sedum_Unigene_BMK.44870 | 0 | gp4 | 127 | middle |
| 30 | 47 | PC-5p-713544_11 | GCGGGGAAACGGCTGGGAAACGGC | 24 | Sedum_Unigene_BMK.60983 | 0 | gp4 | 207 | middle |
| 33 | 50 | PC-3p-125533_95 | TTGCACTGACCGTCGTAGTCGC | 22 | Sedum_Unigene_BMK.80755 | 0 | gp4 | 102 | middle |
| 35 | 52 | PC-5p-222566_46 | AGCTGCTTTTATATGGATCCC | 21 | Sedum_Unigene_BMK.57127 | 0 | gp4 | 178 | middle |
