# Supplementary material for: Integration of small RNAs, degradome and transcriptome sequencing in hyperaccumulator Sedum alfredii uncovers a complex regulatory network and provides insights into cadmium phytoremediation
Source: Plant Biotechnol J. 2016 Jan 23;14(6):1470–83. doi: 10.1111/pbi.12512 (PMC5066797; doi:10.1111/pbi.12512)
Supplement: Supplementary file 13 — File S1 miRNA and their targets names, sequences and primers for RT‐qPCR. [file PBI-14-1470-s009.docx]

1. mtr-miR166C-2SS20TC21CA TCGGACCAGGCTTCATTCCCA

BMK.53452: GRF1-interacting factor 3-like (GIF3)

GCAACCGCCGTATGCTCCGATGACGAACGCGCTTCCGACTGCGCCTCTGACGACCGAGCAGATTCAAAAGTTACTTGATGAGAACAAGCAGCTGATCATGATGATATTGGAGAACCAGAGCCTTGGGAAACTTGCTGAATGTGCGCAGCTCCAAGCATTGCTTCAGAAGAATTTGATGTATTTAGCCGCTATCGCGGATGCTCAACCACAAGTCCAAACTCAGCCTCAAGCAGTGCTTTCTCAGACACCGATCCAACCAGCTCTTCAAGACCGTTTAATGCACCACCCTCAACCGATGATATCTCAACAACAACAGTCTAACATGTTGGCATCAAATATCCCTTCACAGATCAACAACCAAATGCAGCTAACTCAGCCTCAATTACAACAACAGCTGCAGCAACAAGAACAACAAGCACAGTTCCTACCTGGGAATTTAGGTGTTAGACCTCGTAACCAAACTCAGCTGCATCAATTCACACCAAGTAGGCTACTGGATCCAAGTAGTTTCAGCAGTGCATTAGGAAGCCAGCACAATTACACAGGTCCACATGGAAGTGATAGTACGGGAAGGAAGCAGGTTGGCTCTGATAAGTAAGTACTATCATATGTCAACATTAGTGCCACCTCCTTTCCTTTCAAATTTCAATACTCATTTCACCCGAGAGAGTTGCTTCGCTACTGATAAAACAAAACAGACAACATGGATTTTATGATGTTGAAATTGAAGTACAACAGACAGAGATGGAAAGTTCTTAAGCCAATAACGAAATATGCAGTATCCTCGTATCGCATAGGCGATGGCAAGCGCAGTTAAGGTTTTACAATTGCCAACTTCACTATGGTTGATACCTTAGTAAATGTGATGTGACGTTAGAGGACAGCTATGAGATGAGTTTGGGGCCTAATGATCACCTTCCGCTAGAGCTTAAGAAAATAAAGCTTGAACTGCTTCAACCCTCTTTTCGTAGCAAGTTTCAAGGGAATGAAGCCTGGTCCGAGAGAGAGAGAGACTAATGTTTCCTTGAACCAGACATCATTCCCCTCAAGCCTACATACATCGGTGAATCAAATAGCTACAAGGTTGATCATACAGTGTTTTATAGAGAAAGATAGTTTTATACCTGTGTTTTGATACTGTTGCTACTTCACG

GIF3-F 5’- GTCCAAACTCAGCCTCAAGC-3’

GIF3-R 5’-GTTGCTGCAGCTGTTGTTGT-3’

BMK.62570: class III HD-Zip family protein (HD-Zip III)

GTTTCTGTCTAAGGCTACTGGAACTGCCGTGGAGTGGATCCAATTGCCTGGAATGAAGCCTGGTCCGGATTCCATTGGAATCGTAGCTATATCACACAGCTGCACTGGAGTTGCAGCACGTGCGTGTGGCCTTGTGGGTCTTGACCCTCCAAGAGTAGCAGACATCTTGAAGGATCGCCCATCATGGTACCGCGATTGTCGAACAGTTGATGTTGTGAATGTAATGTCCACCGCCACTGGCGGAACCATTGAACTGCTCTATATGCAGCTTTATGCACCTACTACATTAGCTGCAGCTCGTGACTTCTGGATGCTTCGCTTCACGTCTGTTATGGAGGATGGTAGTCTAGTGATCTGCGAGCGGTCACTAAACAACACTCAAAATGGTCCAACTATGCCACCTGTTAGTCACTTTGTTAGAGCAGAAATGCTGCCGAGTGGTTATCTTATTCGACCTTGTGAAGGTGGTGGATCTATTATTCATGTGGTTGATCACATGGATTTAGAGGTGTCTAGTGTACCAGAAGTGTTGCGGCCCCTATATGAATCATCCACCTTGCTTGCCCAGAAAACAACCATTGCAGCATTACGGCAGTTGAGGCAGATGTCTCAAGAGGTTTCTCAACCAGCTATCTCTAACTGGGGAAGAAGACCAGCGGCTTTACGGGCCCTCAGCCAAAGGCTAAGCAAAGGATTTAATGAAGCTGTCAATGGGTTTACGGACGAAGGGTGGAATGTTGTGGAAACTGATGGCACTGATGATGTCACTATTGTTGTCAATTCTTCTCCAAGCAAATTGGCTGGAACAAATCTGTACTATGCAACTGGACTTCCATCCTCAAGCAATGCGGTGCTGTGTGCTAAGGCATCAATGCTTTTACAGGATGTTCCTCCTGCGTTATTAATCAGATTTTTGAGAGAACATCGATCAGAATGGGCAGACAGCAGTATAGATGCTTATTCAGCCTCTGCCATTCGGCCAGGTGTATGCGGTTTACCTGAAGCCAGAGTTGGGAACTATGGAAGTCAAATTATTCTTCCACTGGCTCATACTATTGAGAATGAGGAGTTCATGGAGGTCATTAAGCTTGAAAACTATGGTCACTATCAGGATGAGATGATCATGCCGAGCGACATTTTCTTGTTACAGCTATGTAACGGGGTCGACGAAACAGCTATGGGAACTAGTGCTGAACTCATATTTGCACCAATGGATGCCTCCTTTTCCGATGATGCACCACTCTTACCATCGGGCTTCCGCATTATTCCTCTTGGTTCTGGATCAGATCCTTCCAGTCCAAATCGTACGCTTGACCTTGCTTCTGCACTTGAGGCTGGTTCAGTGGGAAGCAGAGTAGCTGGTGATGCTTGTATCCAAGGAAAGACGAAGTCTGTTATGACGATAGCATTTCAGTTTGCGTTTGATTTGCACCTCCAAGATAATGTAATAGCCATGGCTCGACAATACATTCGTAGCATTATATCGTCTGTCCAGAGAGTTGCATTGGCACTCTCTCCCAATGGGTTTGGATCTCATCCTACCATTCGTCGTCCACCTGGTATCCCTGAAGCTCACACTCTCGCTAGTTGGATCTGCCAGAGCTACAGGTCATCGTTGGGTGTTGACCTACTAAAGCCAAGTACTGAAGGAAATGAACCTATTTTGAAGTCCCTGTGGCATCACTCTGATGCTGTCCTGTGCTGTTCGTTAAAGGCTATGCCGGTTTTCACGTTCGCAAACCAAGCAGGAATGGACATGCTAGAGACAACCTTTGTTGCACTTCAAGACATTACACTTGACAAAATATTTGATGAAAATGGAAGGAAGACACTCTGCAACGAGTTTCCGCAGATCATGCAGTTGGGTTTCATGAGTCTGGAAGGTGGCGTGTGTTTGTCAAGTATGGGAAGGGCAGTGTCTTATGAGAGAGCTGTGGCGTGGAAGGTGCTGGATGAAGAAGAGAATGCTCACTGCATCTGCTTCATGTTTGTCAACTGGTCCTTTGTGTGACCTCGTCCGAACACCCCAAATATGCTGAGCATGCACAACTAGGATAATCCTTGTCTCTTAAACATTGTAGTTATCATGTTGTTACACATGTGTTGTTTTAGTTAGTTTTATCATGATCTTATTTATAGTTCTCTACGTGCGAATGAGCGGATGTATGAACTAATTTAGTCAAATAATCCAATTATTAAAACGAGTCAGAGTATGAAAATAATTACGATATTTTGATTTAAGAAACG

HD-Zip III -F 5’-AGCTGTCAATGGGTTTACGG-3’

HD-Zip III-R 5’-TTGATGCCTTAGCACACAGC-3’

BMK.62457: homeobox-leucine zipper protein REVOLUTA-like (HZP)

AGCGATTGAGCGAGCCAGCGGCTTTTCTGTTTCTCTCTCCCTCGAGATCAGTCGTCGTCGTCGTTATTACCATCTTCATCGCCAACAACAGCGTCTGCTCTTTGTGAGATGTATAAGGTTGCCTGGTGGTGCAACGCTACTGATTTGGTTGAAATTCCCGAGCTGTTTGACGCTTGGTGAACCTCGTTGCGGTCGCTGCTTGTTGCTGGTGATTGTTTATATATTTTTTTAAGAAACGTTGAGAGACAATGGCAATGGTAGCAAGTCAAAGAAGGGAGAATAGCGGAGGGAGTATTATTAAGCATCTTGATGCAGCGGGAAAATATGTTCGCTACACAACTGAGCAAGTTGAAGCTCTTGAGAGAGTTTATGCAGAATGTCCAAAGCCTAGTTCTATGCGGAGGCAGCAGTTGGTCCGTGAATGTCCTATCCTTGCTAACATTGAGCCAAAGCAAATCAAAGTCTGGTTTCAGAATCGCAGGTGTCGTGAGAAGCAAAGAAATGAGTCATCAAGATTGCAGGCTGTAAACCATAAACTGACTGCTATGAACAAATTACTGATGGAAGAAAATGATAGATTGCAGAAACAGGTGTCACAGCTGGTTTACGAAAATGGTTACATCAGGCAACAGCTGCAAAGTGCAACTGGAACAGCTGCTGACGTAAGTTGCGATTCCGTTGTTACCACTCCGCAACCTTCTTTAATAGATGCTAGTAATCCTGCAGGGTTGCTTGCGATTGCAGAAGAGACATTGGCAGATTTCCTATCTAAGGCTACCGGAACAGCTGTTGATTGGGTCCAGATGCCTGGGATGAAGCCTGGTCCGGATTCGGTTGGGATTTATGCGATTTCACAAAGTAGTAACGGAGTGGCAGCTCGAGCGTTTGGTCTTGTCGGCCTAGAACCTCCAAAAATTGCAGAGATTCTTAAGGATCGAACGTCTTGGTCCCGAGATTGCCGAAACCTGGAAGTTTTCACATCTCTCCCCACTGCAAATGGAGGAACTATTGAGCTTGTGTACACACAGATGTTTGCTCCCACTATTCTGGCCCCAGCTCGGGATTTGTGGACGTTAAGATACACAACTTTGTTAGAAAATGGCAGTCTTGTGGTTTGTGAAAGATCTTTGTCTGGTTCTGACGCTGCTCCGATTGCTGGTGGCAACTTTGTTAGGGCTGAAATGCTGCCTAGTGGATATCTCATTCGCTCATGTGATGGTGGAGGCTCAATCATACACATAGTTGACCATTTAAATCTTCAGCCGTTGAGTGTACCAGAAGTTCTTAGACCACTCTATACATCATCCAAACTAGTTGCTCAAAAGATGACAATTGCAGCATTGCAATATGTGCGTCAAATCGCTCAAGAGATGAGTGGTGAAGTGGCATATGGCTTTGGGAGGCAGCCTGCTGTTTTGAGAACATTTTGCCAAAGATTGAGCAGGAGTTTCAATGATGCCATCAGTGGATTTAATGACGCTGGTTGGTCTGTGTTAAATTGTGATGGCGCTGATGATGTAATAATTTCTGTGAACTTAACCAAAAGTGTGACTTCACAAACCAACACAACTGGTTCATGTTCACTCTCTGGAGGCATTTTATGTGCAAAGGCGTCTATGCTTCTTCGTAATGTTCTGCCTGCAGTTTTGGTGCGGTTCCTTAGAGAACATCGCTCAGAGTGGGCTGATTACAATGTTGATGCTTATTCTGCTGCATCCATCATGTCAAATACATTTTCTTCCCCCGAGATAAGATCTACAAGTTTCAGTGGAAGCCAAATCATAATGCCTCTGGGACACACCATTGAAAATGAAGAGATGCTCGAGGTTATTCGAATTGAAGGTCATTCTCTTTCGCCTGAACAATCGTATATGTCAAGGGATATACACTTGTTGCAGATTTGTAGTGGGACTGATGAAAATTCAACTGGGGCTTGCGCTGAACTCATTTTTGCTCCAATTAATGATATGTTTCCAGATGATGTTCCTCTATTACCCTCCGGGTTCCGCATAATTCCGCTGGATACAAAATCATCTGATATGCAGAATCAATCGAAAGGATATCGTACTTTAGATCTTACTTCCAGCCTTGATGTCAGCCCAGTGACTGCTCAACCTACAGGCGGCGCTCCGCTTCAAGGCTCGAGATCTGTCTTGACCATTGCTTTTCAGTTTCCATTTGATACTCACTTGCAAGACAGTGTTGCCAATATGGCTCGTCAGTATGTTCGTAGTGTAATTACTTCCATTCAGAGGGTTGCAGTTGCTATAGCTCCTATAGGAATATGCCCCATGGCAGGACCGAAAGTGTCACCTGATTCTCCCGAAGCGCTGACTTTAGCTCAGTGGATCTGCCACAGTTACAGCTATCATGTTGGAGGTGATTTGCTGAGGGCGGAATCTTCCGCTGGTGATATGGTGTTGAAACATCTATGGCATCATCCCAATGCCATATTGTGCTGCTCCATTAAGGCAACTCCTGTTTTGATATTTGCAAACCAAGCTGGACTTGAAATGCTTGAGACTACTCTTGTAGCGTTACAAGATATATCCTTGGTCAAAATATTTGATGAAGCTGGTCGTAAGGCATTGTGTTCCGACTTTGCCAAGATTATGCAGCAGGGCTATGCATACTTGCCCACTGGGATATGCATGTCATCGGTTGGACGTCAAATTTCCTATGATCAGGCAGTCGCTTGGAAAGTGCTGGCTGGAGACGAAGAAACAGTTGAATGCCTGGCTTTCTCCTTCATCAACTGGTGTTTCATCTGAACTCAAAGCTCTGCAAGACCTCTAGAAAGTTTAAAATTAAAGTATCTATTTTGCAAGACAAGTTATTGTATGAAACCTCAAACTTGGTTTTCTAGTGCTGCATGTGCATGATTCTGCGACGGGGCC

HZP-F 5’-CAGGTGTCGTGAGAAGCAAA-3’

HZP-R 5’-GCAGCTGTTGCCTGATGTAA-3’

2. mtr-miR172d-3p_R+1 AGAATCTTGATGATGCTGCAT

BMK.50491：ATP binding protein （ABP）

GAAGCCACTGGATAATATATGCTACGTAAGCCGCCAGGAAGGCACCCAACCTCCCTTGTGCAATGCAGATGACCAACCAGACAATGTTTGGAATGTTTCTTTAAAAGCATGCATCAGTCGTTTGCCTGAAAATGGGTATGGAGCAAATGTGAGCACTTGGCCCGCACGCCTTAACAATCCACCTGATAGACTTCAGACCATAAACATAGAAGCGTATATATCCAGAAAAGAGTTGTACAAAGCAGAAAACAGATACGCGGAAGAAATTGTTGGATACCATGTCAAATTAATGCATTGGAAGGAGATTGGTTTCAGGAATGTTTTAGACATGAGAGCGGGTTTTGGAGGATTTGCTGCGGCACTTATCAATCAGAAAATCGATTGCTGGGTTATGAATGTTGTGCCTGTCAGCGGTCCTAATACATTACCCGTCATCTATGACCGTGGGCTTATAGGAGTCATGCATGACTGGTGCGAGCCATTTGATACCTATCCGAGAACGTATGATTTGTTGCACGCATCAGGCCTGTTTTCCGTGGAGCAGTCAAGATGCGACATTGCCACTATTATGCTAGAGATGGACAGAATTCTAAGACCTAGTGGCTACGCATACATTCAAGATTCAGTCACAATAATTTACAAACTTCAAGAACTCGGAAATGCAATGGGATGGTACACAAATATTCAAGACACGACGGAAGGCCCCGAAGCTAGCTACAAGATTTTAGTATGTGAAAAACTGCTTGATGGGAAAAATACTCATCAAGGTAAAGGTAGACAGCACAGAAGACGGCACAAAAGAAGTCACAAAAAAGATCATAACTGACGCCATACAGATCATACTCTCTTCGCGCACACTGGTTACATTTTATAAGCCATTGGTGGCAACTGCAGCATCATCAAGATTCTCACATATATATGAAGTATGCTTTTGTGTGAATCTTGATGATGATGCAGCTGCTATCAACAACTGGAGCAGCTTAGTGTGTATCCATGGAGAGAGAGAGAGCTACAGCTATCAACACATATTTAGATGTGAGAATGAAGATGAGTTGAGAGATGGGGGAGGCTTTTTATATAGAGAAAAAGGAAGTAGAGAGAGAATATATAGTTTTTAAGCTAACTAGATATCTAAAGTGTGAGACTGTGAATGTGATTGTGTGGAACTTTGTAGGTCACATTGGTTACACATCACAATCTTTTAAGGTACAAGAATGATGTCATCAAGCTGATCAAACTTCACTTTCAGCCAGACTTGCACTTTCACATATGTCAATGAAGAGATGAGGAATATTATAAATGCCTCAGAATGGAAAGCAACG

ABP-F 5’- CCGTGGGCTTATAGGAGTCA-3’

ABP-R 5’-CCATTGCATTTCCGAGTTCT-3’

BMK.61091：ethylene-responsive transcription factor RAP2-7-like（ERFc）

CGATCTCGCATCAATCATCGTCATACCAAATAATCTCTTCTTCACGCCAGTCTCTCTCTCTCCCTATCTCTCTGGCTTTGTTTCACTCATTCGCTCAAAATGCCTTCGCCCTGAGAGCCTCCTCATCCTCTGTTTTTTTCTCATTAAAAAATAATACTGATAATCCGTCCCCGAAACCCCCTCCGCCGCCGCCGCAGGATCCATGTTGGACCTTAACATTAACGACGACTGCCACGATTTCACCCATCTTCAACAATCACCAGAACAGCACCTTATTCCATCCCAGACACAAACCGAATCCGCCACATCCAGCTCCTCAATCATCGTCAATGCCACGTCAGCCAGCGATGAAGACTCTGTCTCTGCCACAGCATCCAGAACAGTACCAGTACCTTCCACTCTCTTCTTCGACATCTTGAACACAGATCATCATCATCATCATCAAACCCCAGTTCCTGATTCAGGGAATATTATATTCCCGTCGCTGTCACAAACCGACGCCGTCGTTGTTCCACATCCTCCGTGGCTGAATCTCTCCTCCTTTGTCGACTCTGTTTCCCTTAACCGTTCATCTTCTTCATCATCCGAGCTTTGGAGTTTACACCAGCAGCAGAGTCAGCACAAGCCGGCGGCTAAAAAGAGCCGGCGTGGACCGAGATCGAGAAGCTCTCCGTACCGTGGCGTGACGTTTTACCGGCGAACTGGCCGATGGGAATCTCACATATGGGATTGTGGGAAGCAAGTATATTTGGGTGGGTTTGACACTGCACATACTGCAGCCAGAGCATATGATCGCGCAGCCATTAAGTTCCGTGGAGTTGAAGCTGATATCAATTTCGACTTGAACGATTACGATCAAGATATGAAACGGATGTCGGATTTGAGCAAAGAGGAGTTCGTACACGCTCTTCGTCGTCAGAGCACCGGGTTTGCTCGAAGCAGTTCAAAGTTTAGAGGTGTGATTCTGCAGCAGAAGTACGGCCGGCCTGAATCCATATCTGGACCAGCACTAACTAAAATGTATACTTCATTTCAATTTGATGATTCAATTCTTGTATCCGTGTTCATTTGGAACATGTTTGTGACAAGTGACGATGTTTAAGCGCCCAGTCTCAGTGAAGGAGAAGCTGTAGTCAACATTGACGCTAGCAATTACAGTAGATACATCCAGACATCAAGTGTGGACGGCGCTCTAGATTTGAGCCTTAGCGTCACTCCGGCATCGAATGGTGGTTGCCAAGTTGATAGTGATATCATCCGCATCTCTGGATCATCTGGTTATTCAAAATTTGGAGGACAAAGAAACATGGTATGATGAACCGCAAATGTTGTAAATAGTCTCTGGATAAGTTTGAACTGGACGTCATTGATTGCTTAATGACCTTTCTTGTAGTTCGATAGCTCTGCTTCCTCTGTTCTCCGCGGACAACCATCTCCGCCCGTGTGGCAGTTCGCGCAGCCTTCTGGTTCAGGTCACGTTTCCTTTAGTAAGGTAAGAGATGTTATCGTTCAATTTTTAACACGAGTGTATGTATTTGTAGCTTGTTACCATCGCTCTGTTCGCGCAGAGTCCGACAATGGGAAGAAGAATGGTTGTAGCTAAGAATCCTTCGACAGGATTCCGTGACTGGTCATTCGAAGTGCCAGTAGGTCATGCCGGCAATGTAAGTACTGCCTCAATGCAACCTCTGCCAAGTGCAGCATCATCAGGATTCTCATCATGCTTTAAGCCTATTGCTCCGCCAACACATGCATCCGCTCCACGGTCGTTTAAGCCATCAACCAACGCCGTGTACTACAACTACAGGGACTAAATGCTGCCAACAGCAGTCCAGCACGTAGCCATTTGCTGAACCTATTTACAGTGGAGTTATATGAGGCGTTCGACTCGTTGCTTGCAATGTGTACTGTTTACGTGTATCGTATCATTTGTGTCGATAACCATTTGATGTATCCTGATTGTGGTGTGTGTGGACAGAGCGAGTTGCTTTGAAATTTATTACTCTCTATCTTAATTTCCGTGAAATTTGTACTTTACCACAAACAAAAGGACTGAGGAGTAGTAAAAAAAGTTTTAAAAAAACATGTTTGATGCAAGTGTAGAGTGATTAAGGAGAAGGCG

ERFc-F 5’-GCCCAGTCTCAGTGAAGGAG-3’

ERFc-R 5’-GATCCAGAGATGCGGATGAT-3’

BMK.60321: AP2 domain-containing transcription factor (AP2)

GACATCCGGCCACCAACGACGAATCCACAGCCACATCCCTCTCCGTCGTCGTTGCAAGCTGCGCCAAGTACAGTACTACTTCTGTCTGCTAACAACAAGCAAGCTATATACCTGCAAAAGTTGTACTGTGTAGAACTATAGAACAGAGTGTGTGAGACAAGAGATAGAGAGAGTGGAGAGCTGAAGCTCTGTACTGTACTGTACACACACTATGTGGGACCTTAACGCAGATATGATACTGATCGACGATGAACAAACGGATGAAAATCACGCACATCAGCATCACCGTACGCCACAGCGATGGCAACATCAACGAGGCCAGGTGCTACCGGACGATTCAGAAAAATCCACTGTCGTTCTCAACAATGCGGAATATGAAGACCATTCTAACGGCGTCGTTCCACGCACTTTCTTTGCTTTCGATATCTTCAACTCCTCCTCTTCCTCCTCTCCTTCGCTCCTTCACAACCGCGACATAAACACCGACGAAGTGGCTGGTCCGTCGTCGTTCGGTGGGTTAGTGAATGAGAAGACGATTCCGCCGATTATTGAACCGTTCATCTCTCGATCAGCTTCGACGCCGGATTGGCTGAATCTGTCGTTTTCGGAATCGGTCGAGCCGCATGAACAACCGCAACCGCAGCCGCAGATCGTGTCGGTGAAGAAAAGCCGTCGTGGTCCGAGGTCGAGAAGCTCTCAGTACCGAGGCGTTACTTTCTATCGGAGGACTGGAAGATGGGAATCACACATTTGGGATTGTGGAAAGCAAGTGTACCTTGGTGGATTCGACACAGCTCATGCCGCAGCTCGTGCGTATGATCGAGCTGCTATCAAGTTCCGCGGGGAAGATGCTGATATTAATTTCACTATAGCTGATTATCAGGAAGATGCCACTCAGATGAAGAACCTGAGCAAGGAAGAATTTGTCCACATCCTTCGAAGACAAAGCACTGGATTTGCACGGGGAAGTTCCAAATATAGGGGTGTGACGCTGCACAAATGCGGAAGATGGGAAGCTAGGATGGGTCAGTTGCTTGGAAAAAAGTGAGCATTTTCCATATTTGACCAAACCTATCTTGTTCAAAGTCAGTTTGGTTCGTTGGAGTCAGTTTTAAGGTTAGGTATGTTATTTCTATGAGAGTATGTCGTCCTAGGTTTATGTAACATGTTTCGCCGGAAACGGAAAGAGGTAGAGTTGACAGATGACACTTCTCAATTCTCATGCAATGTTGAGAAGACAATGGTTGTTTGCAGGTACATATATCTGGGGTTATTCGACACTGAAGTAGAAGCTGCAAGGTTGCTTGTTTTATAACCACTATAGCTTTATCGATTTAGTGTCTTTTTCTTTGATATTTATGGACTTCATTGAGTTAGGGTACTTTTAAATTGATTGATGTTCACTGTGTTGATTTCGGTTGTTGATTGATTTATTTTGTCTGAATGTAGGGCTTATGATGTTGCAGCTCTTAGATGTAGTGGAGGTGAAGCAACCACAAATTTTGAACCAAGTACGTACGACGTAGATTTCGGTTCACATATTGTTAAACATGATGCTGGTGGTGGTGACCTTGAGCTGAGCCTTAGCATTGATTCTTCATCCGAGTATAATCCCATGTGTAATGCTTCCACCAGAATCGAGGCCGGGAACGGTCCAAAGATACTTTACTCGACGTCGGCTTCCGGAAGTGGGCAAATCCATGCTCCTTATAGTCAACTATCAATATCAGCCAACTATCCGGGCATCCGTCCAGGCAACTACTTGCCAATGTCGAATGAGGTTCAATCAATGGTAGTACCTTCTTCTTCAAGACTGCCCAACTGGGGATGGCAGCATCCACAAGGCCATAACAGTAGTACCATCTTCAGGCCGCCACCGTTCTCCAGCGCAGCATCATCAGGATTCTCTTTTTCCTCTTCCGTCACCCCAGCCACGTCGATTACACAACTGCAACAAGCACGGCAAAGCTATCCATATCAGAATAGTTTCAACAAACCGTCATAAGAAAGCACGCGTCAAGTACAGTGTTCATACTCCTATATCTCTTTACCTTTTTTCATGTGATCAAGATGATGCCTGTATCCTCAATACTCAGGGCTGCCGTCTTTCATCACGCATCATATATTTTGATTATGTAAATGTTTGTCACGTTTATGCGTACCAAAATTGATCAGATATTTTTTTGTCGGACCTGAACTCCAAGTTTTGTAAGCTTCTATGGTAAGCAAAGAAAAGGTTATAATATCGGTTTCTAGCGTTTGCAGCT

AP2-F 5’- CTCCTTCGCTCCTTCACAAC-3’

AP2-R 5’-CGATTCCGAAAACGACAGAT-3’

BMK.39895: two-component response regulator-like APRR2(APRR2) TATCAAGCATATGCTATGCGGTGCACTTGTACACATCCATCCATAACCCAAAGCTTCTTCTCTCTCTATAAATCTCTACTTCTATTGTTTGACAGTTTCTCCCAGCTCTTGGCTGACTTCAAAGCTTCATGCTACTTCACAAGTGGCAAAACTTTTACTACATGATTCACATATATAAAGCCACACACAACAATAGCAACACCTCCGATCTTCCAATACTTATTCATCTTTCTTTTTTTTACTCTACTCTTCATTAATTGCAGTTTGTAGTAGCTTCTCTCTTGATGGTGAGCACCAGGTTAATTAATTAGTCTACAAAGGTCTTACAATTATGTCTTCATCAATCAATCATGATCACTTATCTAGGGAAATCTATTGAAACTTGACCTATAGAACATCATTGTAGACTTGTCTTAGAGTTTCCTACCCTTGCGATAGATCAATGAAGATAATGGTGGCCTGAAAAGCTATGCCGGAAACCAGCAATGGTGTGCACTAGTAAAGATTTACTCGAATGGAATAACTTCCCTAAAGGACTTAAAGTTCTTGTTCTAGCTAAAGACTCTCATTCCGCTTCTGAAATTACCTCAAAGCTTGAAGAAATGGACTACATTGTATCGACGTTCCATAGCGAGGATGAGGCTCTGTCGTCAATCCTCGAAAAATTTGAGGCCTTCCATGTCGCTATAGTCGAGGTCAGTACAATCGACAACAATGGCCGCTTTAAGTTTCTGAGGATGACGAAGGACTTGCCGACAGTCATTGTAGCTGATGCTCAATGCCTCACCACGACGATGAACTGCATCGCTCATGGAGCTGTCGAGGTGTTAAATAAACCCTTATGTGACGACAAGCTTCGCAACATTTGGCAACATGTCCTGCATAAGGCATATAATGCAGGGGACAGTGCTCTGCCGGAAACGCTTACGCCAGTTACAGACTCGACGATGGTAACTGTAAACGAAGAAATGCAATGCTTGTATGATGATGATGATGATCGTTGTGAATATGATAAGTATCCGGCTCCATCGACGCCACAACTGAAACAAATAAGAACACAAGACGACGATGCGAGTTGGAATGATATGAATAAATGGTCAACTGAAAAAGACAATACAGAGCATCATCATCATCATCATCAAGAATCTAAATCCGTCGAAACTACTTGCTGCAACGCAACTCAACAACTTATCCACGATGGTGGTGCAATCAAAGCAGAGAATGATTCGGCCAATGGGTGCCATCAGAAAAATGCGAGAAAGGAGAGTAAGAAGGCATCGGAAAGTTGTGGTCCTTGCGGACCCAAAACTTATCGCAAGAAGATAAAGGTGGACTGGACGTCGGAGCTCCACAAGAAATTCATACAAGCAGTGGAGCAACTAGGAATTGATCAGGCAATACCTTCTCGAATCCTTGAGCTTATGAAGGTAGACGGACTAACTAGGCACAATGTCGCAAGTCACCTTCAGAAGTATCGAATGCAAAGAAGACACACTTTACCGAAAAGAGATGAAATGAGATCATCACAACCTCTGCGTCCAATGCAAGGTGGTTACTATTACCAACATAAGCCGATCATGACCTTTCCGCCGCATACAGCTTATCATTCTTGTAAGGGCTATCCAGTATGGATACAGCCTGGAAGTCACGTGGCCGGCCTCCAGCCTTGGCCGTGGAGGTCTTACTCAACGTTGCATGCTGAAGCTTGGGGCTGTCCGGTGATCCCATCGCTGCAGAGTTCATATTCATATTATCCGCAAAACATGTCCGGAAGTTATAGCGGAACAGTTACTAACGGACAGGGCTTGGTACAGAGTTTCCGCGACGATCATATTGATGATGAGTTCATAGACGGTATGATAAAAGAAGCGATAAACAAGCCGCCGTGGGTGGCACTACCTATAGGACTAAAGCCACCATCGACGGAGAGTGTACTGGTTGAGCTTTACAAGCAAGGCATCTCCAACATACCTCCAACACACAAAAACACCAACTGATGACGCCCTTGCTTGTACTTTGTCTCTTTAGTTTTGGTTTGAATAAAAACGTATGAGCTTAAAGCCAAAGCTTTGTACAGCAATGGCGAAACTGAGATTACATTTACAGATGTTATTAAATATGTAACAGATTAGACACTCCAAAAAAA

APPR2-F 5’-TAGCGAGGATGAGGCTCTGT-3’

APPR2-R 5’- GGCATTGAGCATCAGCTACA-3’

3. PC-3P-3461203-2 TATGTAGTTTTGGGTGAC

BMK.46596：SBT1 protein precursor（SBT1）

GCCAACTCACAACTCACAACATGTTGCGGATGGTATGGTTTGTATCAGTGACCATTCTGCTACTGATAGGAAGCTATGAGAAATACGCGCTAGTAGAAGGTACGAATCCAACTGCTAAGAAAACTTACATAGTTCATATGGACAAGTCCATTAGGCCAGCTAGTTTGGATAATGAAGTTGAATGGTACGATACTTGCTTGAAATCCGTATCTGAATCCGGAGCATCCGAAATGTTGTATACTTATAATGAAGTCATGACTGGATTCTCCGCAAGAATGACTGATACTGAAGCTGAGTTGCTTAAGAGTCAACCTGGTATTGTGTCTGTCGAGACAGAGACGGTCTACGAGCTGCAAACTACTCGTACACCGATGTTTCTCGGGCTAGTAAGTACCGGTGGCTCGAGTATGTTCACGGAGCCTGACCATGTAAGTAACCTCGTCATTGCTGTGCTTGATACTGGGATTTGGCCTGAGTCAGAGAGCTTCGACGACACGGGGTTTGGACCGGTCCCGAGTACATGGAAAGGAGCGTGCGAGGTAGGGACCAACTTCAGTGCTTCGAGCTGTAACAACAAGCTAGTCGGGGCAAGGTACTTCTTAAAAGGGTATGAAGCTGCGGTAGGAGCTTTTAACGAGACTCTTGAGTCAAGGTCTCCAAGAGACGACAACGGTCATGGGACACATACGGCTTCAACTGCAGCTGGCTCTCCTGTACCCGGAGCTAGCCTTTTCGGATATGCACCTGGAACAGCTCGAGGAATGGCTACCAAAGCGAGGATTGCTGCGTATAAAATCTGCTGGGCCAAGGGATGTTTTGGAAGTGATATACTCTCAGCCATTGACGCAGCTGTAGCAGATGGAGCTAATGTTTTGTCATTATCCATTGGTGGAAGCTCACCAGACTATCCGCGAGACAATGTAGCAATTGGAGCCTTTGCAGCGACTCAACGCGGTATAATTGTATCTTGCTCTGCTGGTAACAGAGGTCCAACTCGTTCAACCTTGTCAAATGTTGCGCCGTGGATGATCAGCGTTGCGGCGAGTACTCTAGACCGTGAGTTTCCGAGTTACATAACACTCGGAAATGGTGACAAATACACAGGTGTATCACTCTCTAGCGGAACTCCATTGTCTCCAACTACATTGGTTCCATTAGTCTACGGAGGAAGTGTCCGCAATGGCTCATTTGGAGACCTTTGCATCCCCGGAAGCCTCAACGCAGCACTCGTGAAAGGCAAAATCGTAGTATGTGACCGAGGCCTGAACTCCAGAGTCGAAAAAGGTCAAGTAGTGAGAGATGCTGGAGGTGTAGGAATGATTCTAACCAACACAGCTGCAATAGGCGGCGAGGTGTCGGCTGAGCCTCATTTCATACCCACTGCAGCCGTAGCCCAAGCCACCGGTGATCTGATAAAAAAGTATATCTTCAACAGCTCTAACCCCACAGCCACGATTATCACCGGTGGGACGAAGTTAGGAGTCCAACCCTCACCGGTTGTAGCTGCATTCAGTTCAAGAGGTCCAAATATCATTTCTCCGAAAATCCTTAAACCAGACTTAAGTGCTCCCGGTGTCGATATTCTCGCAGCATGGACAGGTTCATTAGGTCCAACCGGATTATCATCCGACAGTCGCCGAGTGGCTTACAGCATTCTATCAGGAACTTCAATGTCTTGCCCACATGTATCCGGACTAGCTGCATTGGTTAAAGGCGCACATCCAGAATGGAGCCCTGCAGCTGTCCGGTCAGCACTTATGACGACAGCCTACAACGCGTACAAAAACGGAGGTATCATCCTAGACACCGCCTCCAAAAGTCCGTCAACGCCTTTTGCCATCGGTGCAGGCCATGTCAACCCAATACCAGCGCTCAATCCGGGACTAGTGTACAACAATACCATCCAAGACTACATAAACTTCCTATGCGCATCAAGCTATACTCCAGCTCAAATCAAAATCATCACAAGGTCAGATTTTACTTGCGACTCAAACAAGACGTATCGAGTCGAGGATCTAAACTACCCTTCCTTCGCCGTACCATTCCCACGCAGCACAAATTCCACCACAACGGTAACATACACTAGAACATTAACAAACGTCGGAAATGCAGCTACTTACACTGCCTCAGTCTCATCCGAATCCATGACAGTCAAGATCTTGGTCAACCCAGCAACACTTAGCTTCAGCTCATATAATGAGGAGAAGAGTTACACTGTGACATTTACAGGAGACTCAAAGCCATCAGGAACAATCAGCTCCGGCCGAATAACTTGGTCAGACGGGACACATTCTGTCAGCAGCCCAGTCGGTTTCAACTGGAGTTAGAAACTTAGAAATCAAAAACCACAAATCTACATTTTAATGCTACGTTTTGTAAAACTTGAGCCCATGGCTGAAATCACAAATCAGAATCAGTGTCTACAATTTCAATATTCAAATTGATAGTTGAATTTCAATCAGTAATGTAAAGATCGAAACTTTCGAATATCAACTGCAGATATCTTA

SBT1-F 5’-GTGTCGATATTCTCGCAGCA-3’

SBT1-R 5’-GATGTGCGCCTTTAACCAAT-3’

BMK.39438：cytochrome P450 93A1-like （CYP）

GATGATTTTTTTTCTGTATAAGTGGTAGTGATGCTTGATTCGATGGCACGCTGTATTGAGTATGACTCTGCAGTTATCTGAGTGTGCAGAAGGACTTGCTGATACTTTCAGTACCAACAAACCTCTTCTAGCCCTCATTACAGTTTTCACCATCTTCGTTGCCATATGGATAGTCAGGGCATTGAGTAAGACACCCTTTCGGTTTCCACTGCCGCCAGGCCCTCGAGGCCTTCCAGTTGTGGGATACCTTCCGTTCATCGGAACAGACCTCCATATCACCTTTGCCAAACTGGCTAACCAGTACGGACCAGTTTACAAGCTATGGCTGGGTAATACACTATGCGTCGTGGTCAGTTCACCTGCAATGGTCAAAGAAATAGTTCGAGACAAAGACACTATTTTTAGTAACAGGTATCTGACTATTGCTGGATTGATAGGCACATATGGTGGCTCTGATATCGCTTTGGCAGACTATGGTCCTGACTGGCACAAAATGCGCAAGCTTTTTGTGAAAGAAATGCTTTCCAATGCGAGCATTGATTCTTGTTATGACAAAAGGCGAGATGGAGTTCATATGGGCATTAAACAAGTCTATGGTAACATCGGTAAGCCTGTGGATATAGGCAAGATTCTACTACTGACTATTATTGATGTTGTGATTCGAATGGTATGGGGTGGCTCCATTCTTGAAAAAAATCAAGAAAGCTTAACGGAAGGATTGGACTTGAAACAAAGTTTTGCTGAGTTCATGATGATCTTCGGTTCTCCTAACGTCTCTGATTTCTTTCCTGCATTAGCTTGGCTTGATCTTCAATCTTTAGCGAAAAGAATGAGAAATGTATGCGGCCGCTTGAACAGAATACTCGAAGAAGGCATAAATGAGCGTCGAAACTCGAGTATATCATCCAAGCAAGGAAAGAAAGACTTGCTGCAGAATATTTTAGATCTCCAAGAACTCAAAGATTCCGAGTTTTCTCTAAACATAAATCAACTCATGGGCCTCCTAAAGGACATTGTGATCGGAGGAACGGACACAACAGCTACTATAACAGAATGGGCTCTCACAGAACTTCTCAAGCATCCGGAAATGCTCAGACTAGTCTACGACGAGCTGACTCGAGTCGTGGGACTCGACGCCATGGTTGAAGAATCCCATTTACCAAAACTACATTACCTAAAAGCTGTTCTAAAAGAGACTCAC

CYP-F 5’-GGTCCTGACTGGCACAAAAT-3’

CYP-R 5’-CCCCATACCATTCGAATCAC-3’

BMK.52079：serine/threonine protein phosphatase 2A （PP2A）

AATTTTAACGCCCTCCCTCTTTCACCACACCACTCTCTCTATCCTGAATTCAACAAACCCCTCTTCTCTACAATCCCCACTCCCACAGTTGCTGAACTCGATGGCCGTTCTAAGATCCAATCTCTTCGCTCCTTTCTGATTTGTCTCAAGGTTCGGTTTCTTTGTTTTTCCGGTGTTGTGATTTCTCTTGATCTGTTTGCGTGTTTGAATCTTCTCCAGATCTCAATCCTTTTTTTTTTTTGAAATTAGGGTTTGTTGAGTGTCAAATCGTACTGCTTTTTGATCTTCTTGTTACTCTTTAATTAAGTTGTTTAGCAATTCCACTACTTAAAAATCCGGACACCGGAGCTATGCTTAAGCGGATTCTGAGCAAGCTTCCGCAGAAGACTACCAAATGCGTGGGAGCAGATAGCGAACCAAACAACAGGAGTCGTGGAAGAGGTGGTGATGAAGTGAGATGCACAGGTACAGGAACAGTTGCTTTGAATGGTTTAAAGGTATTCAAAAAGGTATCGGCCGCGATTTTTCCGACCAGCGTCGCGGCTGGGATGGAGCTAGTTGAGCCTCATTTAGCGTTTAATGATGTACCCAATGCTGTCAAGCAGAACTTGTTTTTGAGTAAATTGACGTTTTGTTGCTTGATTCAAGATACAAGAGGGGTTTGCAGTGATTCGGAGGAATATGATCGTAAACGTCAGGCTTTGTTGGAACTTGTCGAATTTGTGTCATCTGGATGTGGAAAATATACTGAAGCAGTAGTGATCGTGCTGTTTAAAATGTGTGCGGTTAATCTGTTTAGAACATTTCCACCGAAGGACCGGTTAGCTTTTGGTGGTGGCGAAAGCGAGAAAGAGGAGCTGGTATTTGATCCGGCTTGGATGCATATACAGCTTGTGTATGATCTGCTACTACATTTTATCAGTCACAACTCGCTCGACGTGAAGGTTTCAAAGAAGTATATAGATTGCTCATTCACGTCGAGGCTTATTGATCTATTCGATTCTGAGGATCCAAGAGAGCGAGATTGTTTGAAAACAATACTTCATAGAATTTATGCTAAATTCGTGGTTCATAGACCATTTATCCGAAAGGCAATAAGCAATGTGGTATATCGGTTTGTTTTTGAGACAGAAAGACATAATGGTGTAGCCGAGTTGTTGGAGATATTTGGTTGTGTTATCAGTGGGTTCAGTTTGCCGCTAAGCGGAGAGCACAGAACGTTTCTTCTGCGAGTGTTGATGCCTCTTCATAAACCCAAATCAATGGGCGCGTATCATCAAGAGTTATCATATTGCATCGTACAGTTTATAGAGAAAGAGCAGAGCTTGGCCAACAGCGTTCTTAAGGGATTACTGAAATACTGGCCGGTAACAAACAGCCAAAAAGAGCTTATGTTCTTGAGTGAATTGGAAGAAGTATTAGAGGTATCAAGCATGGCCGACTTTCAAGAAGTCATGGTCCCCTTGTTCCGTCGAGTAACAAGCTGCCTCACTAGCTCACACTACCAGGTGGCTGAGCGCGCGCATCTCTTATGGAACAACAAGCACATCCGTGATTTTATAATCCCCAATCGTCAGATAATTCTGCCTATAGTCATTCCAGCTCTTGAACTGAACACTGACAGCCATTGGAATCGATCCGTGCTGAACCTGACACTGAACTTGAAGAAAACGTTATGTGAAATGGACACCGAACTGGACTCACCTTGTCAAGATACTAATGAGCAAAACAGTACCAAACCGAGCAAAGATAGCCCCAGCAAAGAAATGCATACAACAGAACTACCTGAATCAGATGCAAATTCGCGATCCATTATGGCGGCTTCCAGATCGTTCGTAGAACCTTTGTCGTGTTGACAATCAACAGAGAAGGTGCATTAGTTTAGGTTCGTGGCCAATCAATGGGAAGTGCTACCGGTTTTATTAACAGTTACAGACTGCTTGTGCTTTGTCCAAACTCCAAACCATGATTGATATGCAGAAGGTATTCAAAACTACATCATCGAAGGACAGGACAACCGACTCAAAGACGAAGATGCATCCAGGCGACTCGAACCCAAGTCCTCTTGGGTCAACGATCAAGATTTCAACTGTTGGACTACGTCCCAAGGTACTTATTGATGATTCCATGAGTTATCCGAACCTTAGCAGCACCAAACCCATCGTTGAAAGGAACTGCAAAACTGGCATTGGGTTTACATGCAACCTCTAGCTGAAGGTTTGTGCATCCATGAAAAGCTTCCCAAGGAGCTATTCAGAAGGATTTTTCTCTTAAGAGGATGAGATGGTTCTTGTAGAGATTGGATCGGTGCGAATGCTCTCAGGTTTTCTTAAGGTGGTGTGCTTCTCCAAGATTTGACTCGGACTCGTTAAATTTATGCTTGTATCTAAAACAAGAGTCTCTTGTGTTTTTCAACATTTTAGTGCTAAAATAGTTTTGGAATAGTGTAACGGTGCAAGACTCGTTAATGTGTACATTTTTAGATGGATGTATACATGCACTATAAGATTA

PP2A-F 5’-CCCTCTTTCACCACACCACT-3’

PP2A-R 5’-TCACAACACCGGAAAAACAA-3’

4. mtr-miR164a_1ss17GA TGGAGAAGCAGGGCACATGCA

BMK.23103 NAC80

GTTCCTCGTCGGGATGAAGAAAACGCTCGTTTTCTACCGAGGAAGATCGCCGAAAGGATCGAAATCGAATTGGGTCATGCACGAATACCGCCTCGAAGGAAAACTATCGCTCCAAAACCTCCCTCCATCCGCCAAGACTGAATGGGTCATCAGTCGGATTTTCGAGAAGTCGTACGGAATCAAGAAGCCGCATTTCACATCACTACTTAGCCCGGCGATGTACCGTACCGATTCAGGTCAGTCAGTCCCTATGTTATCTTCGAGCTCGAAGCCATCGATGCACGTTCCCTTCTTCTCCAGTAGCCAAGATGCATTCATCAACTGCTTCAATACAACAACTCTACTGCCAACCACAGCCCCAACCAACAATTACTCATTGATCAACAACCAAGGAGGCGCGTCACAACTTCATTTCGCCGATCAGAATCTTCCGATCATGGCATCAGAAGGTGTTACAGGTCCAGGTCAATCGTCTTCCATGGGGCCTGTTGACATTGAATGCCTATGGAATTACTGAAATTAATATGCAACTTTATCATTCGGAGATCCATTCTTTGAAGAATTAGAGAATTAATTATAGTCGTAAATCACATGAAATCAACATCATGATAAATACTCCCTCCGTCCTGATATGT

NAC80-F 5’- CCGTACCGATTCAGGTCAGT-3’

NAC80-R 5’- GATCGGCGAAATGAAGTTGT-3’

BMK.54779 NAC92

AACAACAACAACATCTCACTTTGCTCTACACATTTTTTGTTATCGATTTGATCAACTATATTCGATCCGACGACTGTTCTTCATTCGATTCGATTAGCTGTGCTAGTTTTCAATTCAAACAAGTTGAATGGATCAAAGGATGGATGATTCCAAAGATGATCATCAGCAGATTGACTTGCCACCAGGTTTCCGATTCCATCCTACCGACGAGGAGCTCATCACTCACTACCTTTCCAACAAAATCCGCGACGCGTCCTTCGTTTCCCTTGCCATCGGTGAGGCTGATTTGAACAAGTGCGAACCGTGGGACTTGTCCGCGAAGGTGAAAATGGGGGAAGATGAGTTGTACTTCTTCTACCTGCGTGACAAAAAGTACCCGACCGGACAGAGGACTAACCGAGCGACAGAATCCGGCTACTGGAAAGCAACCGGAAAGGACAAGGAGATATTTAGAGGCAAGTCTTTGATTGGTATGAAGAAGACTTTAGTGTTTTATAGAGGCAGAGCTCCGAAAGGTGTGAAATCGAATTGGGTAATGCATGAGTACAGATTGGAAGGTAATCTGTCCCTCCATAACCTCCCCCGATCCGCAAAGACGGAGTGGGTGATCTCCAGGGTTTTTGAGAAGAGTTGCAATGGGAAAATGATGCATCAGTCTCCGGCGGTGGCCGATTTCAGCTCTTTCATGTCACAAACTCAGCTGCCTCCGCTCATGGACTTCTCCCAATCCGACACTAAGTTCAAAAACTTCAATACTAGTGCCTCACACAACGTGCCCTGCTTCTCCAACCACACCAGTCTTCAACAACAAGACTTGATCGATTCATACATCAACTATCCGCTATTCGGACTTTCCGCGAATAATCATAATCCTTTCTTGGGGTTCAGCGAGTCAAATTCAAGTATGTTCATATCTAAAAACAATGACAGCGCTGTGATGAGGTCTTATTTGAATAACAGCGATTGGCCATCAGTGCATTTTAAGTCCGGAAGAGAAATGGTCAACATGAACGGCAGTGATATATCATCGTCTTCACAAATGGAGATGGTTAGAAGTCCGTTTGCGGACGGTTCGGCCTCCGGAGGTGGACCTGTCGATCTGGAGTGTATGTGGACTTATTAGAAACAGAGCAAACAGAGCAGATGAGGTGCTCAGGCTCTGTTAAAAAGACTTACTTATTGATTAAAAATACAGAATTAGAATGCTCTTTAGCGTATTGATATTGTGTAAGCCTGTAAGGACGGATACGGATAACGTACCTATTTTGCTACAATGTTTGGTGTTTTTTTTATCTTAGTGTAAAAATAGTGGAA

NAC92-F 5’-GTCCGGAAGAGAAATGGTCA-3’

NAC92-R 5’-ACAGAGCCTGAGCACCTCAT-3’

5. cme-miR858_L-1R+1 CTCGTTGTCTGTTCGACCTTG

BMK.57978：transcription factor MYB12-like （MYB12）

TCTCTTCCGATCTTCCCCCTACACCACTCTCTTCCGATCTCACTAGCTAGCTAGCTATCTTGCTATCATCCGAAACCAGTTGACATCTTCTATGAACACACATATATGGTTGCTTTAAAAGCTAGAGAAAGGTGAGTGGTGTAAATCTAAGTTATACAAAACCACCTTCGATCTTCATTGAGTAGGAAATGGGTAGAGCTCCATGTTGTCAAAAGGTTGGGCTAAAGAGAGGGCGGTGGACTGAGGAGGAGGATCAGATATTGATCAAGTACATTCAAGACAATGGTGAGGGCTCCTGGCGAGCCTTGCCCAAAAGTGCAGGACTTTTAAGATGTGGGAAGAGTTGCCGACTTAGATGGATCAACTACTTGAGAACAGACTTGAAAAGAGGCAACATAACACCACAAGAGGAGGATCTCATCATAAAGTTGCATGCCACTATAGGTAATAGATGGTCACTGATAGCAACACAGCTACCAGGGCGGACAGACAACGAAATCAAGAATCATTGGAACTCTCATTTAAGCCGAAGCATCAATACATGGCGAAGACCCGCAAACGACACTTTGCCAAAACATATCTTAGACAACCTAGCTAAACTGATGGCCAAACCCGAGCCTACAACCAAGAAGGAGAGTAAGAAAAATGCACCAATGAAAAACATTGCGACCGACCGTGACAACAAGCCACCTATTACACACGAAGAGATTGAAGATAATAATTATGATAAACATACAATGCCGGATATTGGGTTTAAGGAATCATGGAGCACCGATCAGTCTCTCGAAACCATATTCTCATTAGTGGATCATCAAGATCTCGTGGAAGGGTTTGAAGAAGACTATGTGGGTCTACTTTCATTGAATGAGATGGTTACACCTTTAACGGACCAATTGATAGTAATGCCTCATGAGCCTGAACATGTTTCAAATGAATTTCAAAACCTAGACGATGATGGAGGTGGAGCTATTGATATTGAGTTTATGAATAACTTAATGACCAACTTGGGCACTAGCCATGCAGAGAAGGACATGACACTGATGACAATGGACAACGTCGACGACATTCCCACATTAAACCCATCACTACCATCGTCTCCTTACTTCTATGACGATCAAATGATGAACATGATCAATAACGACAAGAGCTGTTGGGACGATTATGGTGAAACCCTATATCAAGACCTGATAAATCATGAAGAGGTCGGCAGCTTCTTGATGCCTTGGCTATGGGATTGTTATGACGTGGAGAGCTCCGGACTGTGAAAAGTGCTACTTTATATATAGCTCAGTTGTTTAGTTGCACTACTACTTTCCGACTCAGTTATTGTTACTTTAATTTGTTATAGTGTACGACAACCTTGTTGTATCTATATGTGTGTCTCATTTGGTTCGTGTCAGTTATTGCATGAGACGACAGTTACTAGCTATCCAGCTAATTTTTTGGAGGTAGTCACTAAATGGGAATCGATCGAATGGTGGTGTAAGTGTAATGTAATATAGAAACATTTA

MYB-F 5’-GATCTCGTGGAAGGGTTTGA-3’

MYB-R 5’-CTCCACCTCCATCATCGTCT-3’

6. bra-miR2111a-5p TAATCTGCATCCTGAGGTTTA

BMK.53458：F-box/kelch-repeat protein（F-box）

CAACCCCATTCTTTCATTTTCAGCTACCCACTTACTTCTCTCTCATCCCTTCTTGCCATCTGTTCTTAAGCTTTCATCTTTCTGGCTTAATATGGAGGCGCTCTGATCCGCAACTGGCTCCGGTCTCTGATCTGGTGTCCAAGGCATCTTACAAATGTGCGGCCAAGAAGGTGAAGAAGTAAGAGGTAAAGATAGCATTCGAATAAGGGGTCAGAATATGGAAGAAGAAGAAGAAGTCTGTGACGACAGCCTCTGCCTGTCATTGGGATTAAGTATCCGCCCGCCAAAAAAATCAAAAATTCGCAAAGTTAATAAGCTAGTAAACCTGAACTTGGATATAAAGACATTACCTCAGGATGCAGATTATAATCAAGTTTCTCCATTGAGCGACGAGTTGGAGAATCTGATCATGGCAAGATCACCAAGATCAGAATATTGGAAATATTGCCATGTAAATAAACGCTGGTTGTCTATGATAAAGAGTGGTGAGGTTTACAAAATCCGTAGAGCTATTGGGTTCAGGGAGCCATCAGTTTATGTATTGGCAGGCGGAGATAGTAGCTGGTGGGAATTCAATAGACTATTTAAGTCATGGCGCAAGCTTCCTATTTTACCAGCGGATTGCATAAGTTTCACTCTTGGGGACAAAGAGACACTTTGTGCAGGTACTCATTTGTTAGTTTCGGGAAAGGAGTTTGATGGCTTGGCTATATGGAGATATGATTTAGCAATGAATAAATGGTACAAGGGTCCAAATATGTTGAGCCTTAGGTGCTTATTTGCATCTGCGACGTGTGGAAATTTTGCGTATGTTGCTGGCGGCATAGGTTCCGACAACAAGATTATAAACAATGCAGAAAAGTATAGCCCCGATACTAAATCCTGGAGGCCTTTACCGGCTATGAATCGTAGAAGGAAATTTTGTTCAGGTTTTTACATGGATAAAAAGTTTTACGTCATTGGAGGGAGGGATGAGAACAACAATGATCTCACTTGTGGCGAGGCCTTTGATGAAACTACAAACACATGGAATCTCATTCCAGATATGCTCCGTGACACTCCAATGTCATCCTCCCAATCACCACCTTTAGTAGCTGTCGTCAACAACGAGCTCTACTCACTCGACACATCCTCAAACGAGCTCAAAATATACTCAAAAAACACCATCAGTTGGAAAAACCTCGGACCAGTTCCAGTCAGAACAGACTTTAACCGTGGCTGGGGCGTCGCGTTTAAGTCCTTGGGAAATGAATTACTTGTCATTGGCGCATCTTCTTCTGTGCCGCTAGCTGGCCATGGAATGAGCATTTTTACTTGCTGTCCTGATCCCAGTTCAAATGAGAAGCTCGACTGGAAGCCACTCGACAGTAGACACGGCCATCGGAGCAGCCATTTCATCATGAACTGTGCCGTTATGCTCACTTAGCTCAAACACTCTGTCCTCTTGTTGTTGTGCAAACTGTAAACTGGCTTTCAGTTTCAGCTTTTCAGGAATTTTTTTTTAACATTATAACCCGTCGGTAATTGTACACTTTCGTTGCTAAATGTGTTGCACGCGTGACGCGTCAGACTTTAAAAAAAACAACCCGCCCGC

F-BOX-F 5’-TAGCGAGGATGAGGCTCTGT-3’

F-BOX-R 5’-GGCATTGAGCATCAGCTACA-3’

7. bra-miR162-3p_1ss8AG TCGATAAGCCTCTGCATCCAG

BMK.48574：endoribonuclease Dicer homolog 1-like （DICER）

CTTTGTCCCGAATTTATGATGGTAAAACATGAAAAATCTGGAGGACTTGTGGAGTATGCATGCAAGCTTCAACTTCCATGTAATGCACCATTTGAAAATCTTGAGGGACCACTGTGCAGTTCACCGCGTCTTGCACAGCAGGCAGTATGTGTTGCCGCCTGCAAGAAACTTCATGAAATGGGTGCATTTACGGATATGCTTTTACCAGATAAGGGGAGTAGAGAGGAGGGGAAGTCCAACCAGGGTGATGAAGGAGATCCACTCCCGGGTACTGCTAGACATAGAGAATTCTATCCAGAGGGAGTAGCTAATGTACTGCAGGGGGATTGGATATTATCTGGAAAAGAAGGTTGCTCATACTCCAATCCAGTTCATGTTTACATGTACGCTATTCAGTTCGAGAACATTGGTTCATCAACTGATGCGTTCATTAATCATGTTTCGGAGTTTGCAATATTGTTCGGCCAAGAGCTGGATGCAGAGGTGTTATCGATGTCGATGGATCTCTTTATCGCTCGGACCATGAAAACTAAGGCATCTCTTGTCTTTCAGGGCACCTTAGTTATTACTGAAAGTCAGCTGGCGTCCCTTAAGAACTTTCATGTGAGACTAATGAGTATCGTGTTGGATGTGGATGTTGAACCTGCAACCACTCCATGGGATCCAGCCAAGGCATACCTGTTTGCGCCTGTGGTTAGCGATAAAAAGATGGATACTAGGAAGCATATTAATTGGATGCTAGTGGAAGATATTGTCAAAACAGATTCGTGGATCAATCCCCTTCAGCAAGCTAGGCCTGATGTTTTCCTTGGCACAAGTGAGCGGACTCTAGGTGGAGATCGCAGGGAGTACGGATTTGGAAAATTGCGGCATAGCATGGCATTTGGGCAGACGCCTCATCCGACATATGGAATCAAGGG

DICER-F 5’-AGAGCTGGATGCAGAGGTGT-3’

DICER-R 5’-TGCAGGTTCAACATCCACAT-3’

8. cpa-miR167d_R-1 TGAAGCTGCCAGCATGATCTG

BMK.38910 pentatricopeptide repeat-containing protein (PPR)

TTTCGCTCCTGGGTCGTGCTGGCAAGCTTCAAGAAGCACATAACTTTATTAAGCAAATGCCAATTCAGCCAGCAGCAATAGTGTGGCGCAGTTTGCTCAGCGCTTGTTGGGCCGCTGGGAACTCTGAGTTGGGTGTGTATGCTGGAGAGATGGCGATCCACTGTGATCCATATGACAGTGCTTCCTATGTCTTGTTATCTAACATTCTAACATCTGGAGGGAA

PPR-F 5’-GGCAAGCTTCAAGAAGCACA-3’

PPR-R 5’-ATCGCCATCTCTCCAGCATA-3’

9. han-miR3630-3p_L-1_1ss2GA ATGGGAATCTCTCTGATGCTT

BMK.21215 amino acid permease 3 (AAP3)

TCCCACCTTCTCATACATCACCTTCTTCCAAGTCTAACTCGACTTCTTTGTCATCAGTTAGTAAACTAAAAATGGGTGTAGCTGACTTGGAAGCTAGAAACGGCCACTATAACAACCGAGTCTTCGAGGTTGATCCTTATAACGGGTCTGAATGTTTCGACGATGATGGCCGCATTAAAAGAACTGGTACGTTTTGGACGGCGAGTGCGCATATTATCACAGCAGTGATCGGGTCAGGGGTTCTGTCCTTGGCTTGGGCGACAGCGCAACTGGGATGGGTTGCAGGCCCTGCTGTCTTGTTCTTATTCTCATTTGTCACTTACTATACTTCAACACTGCTTGCTGTTTGCTACAGAAGTGGCGACTCTGTCAATGGAAAGCGTAACTATACGTACATGGATGCTGTCAGATCGTATCTTGGTGGATCTAATGTGAATTTCTGCGGATGGATTCAGTATTTGAACTTGTTTGGAGTTGCAGTTGGATACACTATCGCATCCTCCATCAGCATGATGGCTATTAAGAGGTCAAACTGCTACCATGCCAGTGGAGGAAAAGACCCGTGCAAGATCAACAGCAATCCATACATGATTGGATTTGGAATAGCTGAAATTTTCCTGTCTCAAATCCCGGACTTCGACCAGCTCTGGTGGCTATCCACGGTGGCTGCTGTCATGTCCTTCACTTACTCAACAATCGGACTTGGACTCGGTGTTGCCAAAGTTGCAGAAACTGGGAAGATTCGTGGCAGTCTCACCGGAATAAGCATTGGAACTGTGACTGAGAGTCAGAAGATATGGAGAAGCTTCCAAGCCCTCGGAAACATCGCGTTTGCCTACTCGTTCTCAATGATTCTGATAGAAATTCAGGACACACTGAAGTCTCCACCATCAGAAGCAAAGACGATGAAGAAGGCAACTTTGTTAAGTGTTGTGGTCACAACTCTGTTCTATATGCTGTGTGGCTGCTTTGGTTATGCTGCCTTCGGAGACTTAGCCCCCGGGAATCTTCTAACGGGTTTCGGATTCTACAACCCCTTTTGGCTACTTGATATCGCCAACGTCGCGATTGTGATCCATTTAGTAGGCGCATACCAAGTCTTTTGCCAACCCTTATATGCTTTCATTGAGAAAAAGGCAGCTGAGAGATTCCCAGAGAGCGACTTCATTACCAAGGACATCCTTGTGCCCATACCGGGCTTTAATAAGTCTTACAAACTCAACCTCTTCCGATTGGTGTGGAGGACGGTCTTTGTCATCATAACTACTCTAATCTCGATGCTACTCCCATTTTTCAACGACGTGGTTGGCCTGTTGGGCGCTCTTGGATTTTGGCCGCTGACAGTTTACTTTCCGGTGGAGATGTACATTGCTCAGAAAAGGATCCCAACATGGAGCACTAGGTGGCTTTCATTGCAAATATTGAGCATGTCTTGTCTTGTAATAACTATTGCCGCCGCAGTTGGATCCATCGTCGGGGTTGTTTCCGATCTAAAAACTTACAAGCCATTCAGCACAAGTTACTGATTCGGCAACAAGCTAATGCTTAGCATGGACAAGTTGGAGGATGTTACACAATTCCGGAATCAGATAGCTCTGAAAGTTAGGACTATGTAACCGTCTTTGCTTACGTTACTCAGTTGTTGTACAATCATAAGATCATTTTCTGTACTGTATTACTCAATGAAGAGAAGCAGAGCCGCAAGGTGATGCAATAATCATGTACTTAAATCTTCAAGGAATTGGATCTCATGCTGTCAATCGCCATTGGCAGCAAGATAAAGTGATGTGTTTTGATAAAATAAATGGTAAAATGACTACGATTCCAATCTTCGACGCATCTTTTAAGTTTTTTTGTCACCGGGAC

AAP3-F 5’-GCGTTTGCCTACTCGTTCTC-3’

AAP3-R 5’-AGTCTCCGAAGGCAGCATAA-3’

10. mtr-miR2592bj-p3_1ss12TC ATTCCCACTGTCCCTGTC

BMK.61331 ATPase family AAA domain-containing protein 1-A(ATD1A)

TCACCAGCAGCAATTAACGGATCTCATCTACCATCAGTTCAAACGCATGCCTCTTTGATTGACGTCATTACTCGGCCTTGGCGGTTTCGTGTATCCTTGGATTTAGAGATATTGAAGTTGCGATGTATGTGAGTCGTTTAAGGTGTAGAAATCGAAAATGGAATGTTCTGGCGCAGACTTCGCTGTGTTGTGGTAGGCAGGGTTATCATCAACCTGTGTTGCGTGTTCAGTCTCTGAATCCAGCTACGACGGTAAAAACGTATGTATCGCAGACATTATTATCACGCAGTGCAACTTTGAGAGCTAATAATTTGTGGCTCCGTGAGAATGGAAAAGCGTGTGTTATCAGTAGTTTAGATAGATTGCGGTTATTCAGCTCGGAAGGGGATGGTGGGAATGAAAATGAAGCGATACCAGTAGTGAAAGAGGCGGAGAATGTTGAAAAGAGGAATGATACAAAGGAAAAGGTTAATCAAAATTCGAGGCATTACAATGCTCATGCAAGACTCGGGGAGCAAGATCAAAAGGATTGGCTGTTAAATGAAAAGTTGGCGATTGAATGTAAAAAGAAAGAGTCGCCTTTTCTTACCAGACACGAGAGGTTTAAAAATGAATTCTTGCGGAGGGTTGTTCCATGGGAGAAGATAACTGTATCATGGGATTCATTTCCATACTACATCAATGAGACCACCAAAAGTCTTTTGATAGACTGTGCCGCCTCTCATATAAAGCAAAAAACATTTGCTCAATCTTTTGGTACACACTTGACTTCTTCGAGTGCGAGGATACTTCTTCAGAGTGTTCCAGGCACCGAGCTTTATCGGGAGAGATTAGTTAGAGCACTAGCGAAAGATCTCCAAATTCCATTGCTTATCTTGGATAGCAACGTTCTTGCTCCTTACGACTTTGGTGAAGATTATGATTCAGAAAGTGAGTCTGAAGACGAAGATCATGTTGGGGAAGAGTGTACAACGGAAGAGGATGAAGATGAGAATGATGCAAGTAATGGAGAAGATTCGACGGGTAACACTGAGAAGTCTGGCAGCGATAATGAGGTCAACGTGGAGCTGACGGAAGAAACACTGAAAAAGTTCATTCCCTTCAATATTGACGATTTAGCTAAGCAGAACCTTTCTGGTGAAACTGAAACTTCCTCGCAAGCTGCTGGATCTGCCGAATCCTCTAGCATAAATATAGGGTCACTAAAGAGAGGTGATAGAGTAAAATACACCGGGCCTACCATTCGCATCGAAGCTGACAACAGGATAAAACTGGGGAAGATTCCGACTTCTGATGGATCAACAATTGCATATACTACTATTCGACAGAGATCTTTATGCAGTGGTCAACGAGGAGAAGTATATGAAGTTAATGGGGACCGTGTTACAGTAATAGTGGATGCAAGTGAGAACAAAATTGAGCAAGATGCTGATAAACCTAAGAAAGAAGCTGTGACACCCTCTGTGTATTTAATACATGTTAAGGATTTGGAGCTTGATCCTGAGGCTAGGGCTGAAGATTGTTATATAGCAATAAAAGCGTTGTGCGAGGTTTTGCAGTCCCAACAACCAATGATAGTGTATTTTCCCGATTCTTCTCAATGGTTGTCGAGGGCTGTTCCAAAATCTAACCGGGCAGAATTTGTCCAGAAGGTGCAAGAGCTGTTTGACCAATTATCTGGCCCCGTTGTCTTAATTTGCGGCCAGAATAAAGTTGAAACAGGGTCCAAGGAAAAGGAGAAATTCACGATGATCCTCCCAAATCTTGGTAGACTTGCTAAGCTGCCTCTCTCCTTGAAACAACTGACTGAGGGACTCAAAGACACAAAGAAACCTGAAGATACTAAAATATCTGCCCTTTTCTCCAATGTGTTCTCAATACAACCTCCAAAGGAGGATGATCTGCTGCGTACTTTTAATAAGCAGGTTGAAGATGATAGAATAATAGTAATTTCTCGAAGCAACTTGAATGAACTTCATAAGGTTCTCGAGGAAAACACACTTATGTGCATGGATTTACTACATGTAAATACTGATGGTGTCATACTAACGAAACAAAAGGCAGAGAAAGTTGTTGGATGGGCTAAAAACCATTACTTAGCCACATGTTTACTTCCTAACGTTAAGGGAGACAGATTGTATATTCCTCGTGAAAGTCTGGAGATTGCGATCAAAAGGTTGAGGGACCTGGAATTACTGTCCAAAAAGCCATCACAAAATTTTAAGAATCTTGCCAAGGATGACTACGAGAACAACTTTGTATCTGCCGTAGTTCCAGCAGATGAAATTGGTGTGAGGTTCGATGATATAGGTGCTCTTGAAGACGTCAAGATGGCACTAAATGAACTTGTCATTCTGCCTATGAGGAGACCTGAGCTGTTTTCCCGTGGAAACCTTTTGAGGCCATGCAAAGGAATATTGCTTTTTGGACCTCCCGGAACTGGGAAAACACTTCTTGCGAAAGCCTTGGCCACAGAGGCAGGAGCAAATTTTATCAGTATAACAGGTTCAACTCTTACATCAAAGTGGTTTGGAGACGCAGAAAAGCTCACCAAGGCATTATTTTCATTCGCCAGCAAGTTAGCACCAGTTATTATCTTTGTGGATGAGATTGATAGTTTGCTTGGTGCTCGCGGTGGAGCGTTCGAGCATGAAGCAACCAGAAGAATGAGAAACGAGTTCATGGCTGCATGGGACGGATTAAGAACCAAAGACAGCCAAAGAATTCTCATTTTAGGTGCTACAAACAGGCCCTTCGATCTTGATGATGCCGTGATACGTAGACTACCAAGGAGGATTTATGTTGACTTGCCAGACGCTGCGAATCGCGAAAAGATCCTCAGAATATTTCTATCTCGAGAGAACTTGGAATCTGGTTTCAATTATGATAAACTCGCAAAGGAAACTGAAGGATACTCCGGCAGTGATCTGAAGAATCTGTGTATAGCTGCTGCATACCGACCTGTTCAAGAACTCCTCGACGAGGAAAAGGGAGGCAAAAGCGAGTCTTCATTGCTGAGACCGCTTAACTTAGAAGACTTTATCAAAGCGAAATCAAAGGTTGGACCATCAGTAGCATACGACGCGACGAGCATGAATGAATTGAGAAAATGGAATGAACAATATGGTGAAGGTGGAAGTAGGAGAAAGTCTGTATTCGGATTCTGAAGCAATCTGAAAACGAGTGTTTTTTTACTTGCGGCCGCGGCGGCGACGATGCCAACAACATGGGAGAACACCGGAATGTGTAGAATTAACAACAGGCGTATCATTTGTTATAAATTATAGCATCTCCTTGATGGTTTTCGTTATAGCGTTAGAGTATACATTTTTGAAACATTTTGGTAGAACATGTTTTTGTTCATTTCAGATGTTCTCTATGAATAAAAACACGTTTCGTGTTATTGCGATTGTTTTGTTAGCAATAATTAGAATTAGGGTTTGTTTGG

ATD1A-F 5’-CGACGAGCATGAATGAATTG-3’

ATD1A-R 5’-CGGTGTTCTCCCATGTTGTT-3’

11. rco-miR535 TGACAACGAGAGAGAGCACGC

BMK.57900 MATE efflux family protein 4 (MATE4)

ATGATGATGATGGTGAGGATGAGGGTTTGATGGAAGTGGCAATGGAGGCTGAAATGGGAAGCCAAAGCATTTGGAGTGACATCAAAGAGATTGTGATGTTTACTGGACCTGCAACAGGGTTATGGCTTTGTGGTCCTTTAATGAGTTTGATTGATACTGCTGTCATTGGTCAAGGAAGTTCCATTGAACTTGCTGCTTTAGGTCCTGGAACTGTGTTATGTGATTACATGAGTTATGTATTCATGTTCCTTTCAATCGCTACTTCAAATATGGTTGCTACTTCACTCGCTAAAAAGGATAAAGGAGAGGTGCAGCATCAGATATCAATCTTACTTTTTGTAGGACTTGTGTGCGGTGTTTTTATGATGATCTTCACGAAATTCTACGGTGCTTGGGCTCTCGCAGCTTTCGCTGGCCCCAAAAATGCACACATTGTACCTGCAGCGGACATTTACGTTCAGATACGAGGTTTAGCGTGGCCTGCAGTTCTTATCGGATGGGTAGCTCAGAGCGCAAGTTTAGGGATGAAAGATTCATGGGGTCCTTTAAAGGCTCTGGCGGTCGCTAGCGTTGTAAATGGCGTCGGTGACATAGTTCTCTGCAGTTACTTGGGTTATGGTATTGCTGGTGCGGCATGGGCTACTATGGTATCACAGGTTATTGCGGGTTACATGATGATTCAAGCATTAAATGATAAAGGATACAATGCTTTCGCTTTCTTAGTTCCATCGTTAGTTGAGCTTCTGCAAATAGTTGCACTGTCCGCACCAGTGTTCGTTACAATGATGTCCAAGGTCGCCTTTTACACGCTTATAATATACTTCGCGACATCCATGGGGACAAACACAGTTGCTGCTCATCAGGTTATGATTCAGACGTATACAATGTGCACAGTTTGGGGAGAGCCACTTTCGCAAACAGCTCAGTCATTCATGCCGGAGTTACTCTACGGCGCTAAGCGTAGTTTGCCAAAGGCCAGAAACCTGTTGAAATCACTTCTTATCATCGGCGCTACGGTTGGTTTAGTCTTGGGAACGATCGGGACGTTAATTCCCTGGTTGTTCCCTTACATATTTACGTCGGATCCGGCTGTGACTAAACAGATGCATCAAGTTTTGGTTCCATATTTTCTCTCGTTGTCAATCACGCCATGTACTCACTGCCTCGAGGGATCCCTCCTGGCTGGAAGGGAACTAAAATTCTTGAGTTTATCGATGAGTGGATGTTTTACTCTGGGTGCACTTTTGCTCGTGGTTGTTGGCAGTAAAGGATTTGGCTTGCCAGGCTGCTGGTATGGCCTAGTAGGTTTTCAATGGGCAAGATTCTTCCTCGCACTTCAACGACTGTTCTCCTCCAATGGCGTGTTAATGTCAGATCGGCAGGAGCAACGACAATTGGAAAAAAAGCTGAAAGCTGCATAAGTCCAGGACATGAACAACTGCATTTTAAGCTGGTGAAGTTGTTGTGATTCACTTTAAATTCATGTAATCATTCTTTACTCGCACAAGATATATTCTACGTAAGACAAATATTTGAAATTTGATTTGATAAAT

MATE-F 5’-TACGGCGCTAAGCGTAGTTT-3’

MATE-R 5’-GCGTGATTGACAACGAGAGA-3’
